# Diversity analysis of the rhizospheric and endophytic bacterial communities of *Senecio vulgaris* L. (Asteraceae) in an invasive range

**DOI:** 10.7717/peerj.6162

**Published:** 2019-01-07

**Authors:** Dandan Cheng, Zhongsai Tian, Liang Feng, Lin Xu, Hongmei Wang

**Affiliations:** 1 State Key Laboratory of Biogeology and Environmental Geology, China University of Geosciences (Wuhan), Wuhan, China; 2 School of Environmental Studies, China University of Geosciences (Wuhan), Wuhan, China

**Keywords:** Endophytic bacteria, Invasive plant, Bacterial community, 16S rRNA gene, Plant–microbe interactions

## Abstract

Increasing evidence has confirmed the importance of plant-associated bacteria for plant growth and productivity, and thus it is hypothesized that interactions between bacteria and alien plants might play an important role in plant invasions. However, the diversity of the bacterial communities associated with invasive plants is poorly understood. We therefore investigated the diversity of rhizospheric and endophytic bacteria associated with the invasive annual plant *Senecio vulgaris* L. (Asteraceae) based on 16S rRNA gene data obtained from 57 samples of four *Senecio vulgaris* populations in a subtropical mountainous area in central China. Significant differences in diversity were observed between plant compartments. Specifically, the rhizosphere harbored many more bacterial operational taxonomic units and showed higher alpha diversity than the leaf and root endospheres. The relative abundance profiles of the bacterial community composition differed substantially between the compartments and populations, especially at the phylum and family levels. However, the top five phyla (Proteobacteria, Firmicutes, Bacteroidetes, Actinobacteria, and Acidobacteria) accounted for more than 90% of all the bacterial communities. Moreover, similar endophytic communities with a shared core set of bacteria were observed from different *Senecio vulgaris* populations. Heavy-metal-resistant, phosphate-solubilizing bacteria (*Brevundimonas diminuta*), nitrogen-fixing bacteria (*Rhizobium leguminosarum*), and cold-resistant bacteria (*Exiguobacterium sibiricum*) were present in the endosphere at relatively high abundance. This study, which reveals the structure of bacterial communities and their putative function in invasive *Senecio vulgaris* plants, is the first step in investigating the role of plant–bacteria interactions in the invasion of this species in China.

## Introduction

The advent of globalization has increased the frequency of invasive species outbreaks (Hulme, 2009). Invasive plants can displace native species, destroy the structure and function of local plant communities, and influence the various animals or microbes inhabiting local communities, leading to decreased local or regional biodiversity and, ultimately, an unbalanced local ecosystem and loss of ecological function (Pysek et al., 2010; Blackburn et al., 2011). There are concerns that the constant expansion of invasive plants reduces the uniqueness of local flora and even leads to the global homogenization of species composition (Orians & Ward, 2010). To control the invasion of exotic plants, it is essential to understand the mechanisms of the invasion process; accordingly, this topic has become one of the core research areas of invasion ecology.

Many studies of plant invasion mechanisms have focused on the relationship between plants and macro-organisms that are natural enemies or competitors of plants (Blossey & Notzold, 1995; Keane & Crawley, 2002; Müller-Schärer, Schaffner & Steinger, 2004; Joshi & Vrieling, 2005; Callaway et al., 2008). However, plants can also form mutualistic relationships with microorganisms. Land plants are colonized by microbiota in the rhizosphere, phyllosphere, and endophytic compartments (within the leaves and roots) (Haichar et al., 2008; Rodriguez et al., 2008; Bulgarelli et al., 2012; Lundberg et al., 2012). It is well known that arbuscular mycorrhizal fungi and root nodule bacteria form mutualistic symbioses with plants (Hardoim et al., 2015). Moreover, it was recently recognized that bacteria other than rhizobia (bacteria that fix nitrogen after becoming established inside the root nodules of legumes) are beneficial to plants. Such plant growth-promoting bacteria (PGPB) or plant growth-promoting rhizobacteria (PGPR) can stimulate plant growth, increase yield, reduce pathogen infection, and reduce biotic or abiotic stress without conferring pathogenicity (Compant, Clément & Sessitsch, 2010; Pieterse et al., 2014). Many PGPB and PGPR can produce growth-promoting substances, such as indole acetic acid (IAA), gibberellin A3, zeatin, and abscisic acid (Perrig et al., 2007). Many nitrogen-fixing bacteria, in addition to *Rhizobium* species, have been identified from plants (Gaby & Buckley, 2011).

More generally, endophytic microbiota include all microorganisms that colonize internal plant tissues for all or part of their lifetime, regardless of whether they form pathogenic or mutualistic relationships with plants (Hardoim et al., 2015), and many members of the endophytic microbiota do not cause plant infections (Rodriguez et al., 2009). Some PGPB are endophytic microbes that can enhance the tolerance of host plants to stressful environments, promote plant growth, and improve plant protection (Bulgarelli et al., 2013). Moreover, unlike PGPR, endophytic PGPB can be propagated to the next generation of plants by seeds (Truyens et al., 2015). Accordingly, it can be assumed that endophytic bacteria can establish long-term symbiotic relationships with host plants and thus have an evolutionary impact on the adaptation of plant populations.

In recent years, several studies have suggested that endophytic bacteria play an important role in plant invasion mechanisms. *Sorghum halepense*, an invasive plant that thrives on low-nitrogen grasslands, contains endogenous nitrogen-fixing bacteria that improve the availability of soil resources (Rout & Chrzanowski, 2009; Rout et al., 2013). The effects of rhizo- and endophytic bacteria on the invasion of exotic plants are species-specific and vary across environmental conditions (Long, Schmidt & Baldwin, 2008; Rout & Callaway, 2012; Dai et al., 2016). While previous studies have been conducted to investigate fungal diversity in invasive plants (Shipunov et al., 2008; Mei et al., 2014), it is equally important that the bacterial diversity associated with invasive plants is explored to understand the plant–bacteria interactions that occur in the invasion mechanisms of alien plants.

*Senecio vulgari*s (Asteraceae), an annual or biennial herb, is considered as a weed in the United Kingdom, Western Europe, North America, Australia, and New Zealand (Paul & Ayres, 1987; Müller-Schärer & Frantzen, 1996; Vitousek et al., 1996; Frantzen & Hatcher, 1997; Robinson et al., 2003; Figueroa et al., 2007). It is a small plant with a short life cycle and a high self-crossing rate. It can produce large numbers of seeds, which can germinate under the right conditions at any time; therefore, its ability to spread is very high (Robinson et al., 2003). This species was introduced into northeast China in the 19th century, and it is now widely distributed across China and is included in The Checklist of the Invasive Plants in China (Ndihokubwayo, Nguyen & Cheng, 2016; Zhu et al., 2016; Cheng et al., 2017). *Senecio vulgaris* grows well in ambient habitats, such as gardens, lawns, and arable land, and also survives in stressful habitats such as roadside areas and waste facilities (Robinson et al., 2003). Similar to many other invasive plants, *Senecio vulgaris* disperses along motorways in China (Tian et al., 2018). The top soil along roads in cities, as well as near highways or railways, is commonly contaminated by heavy metals released from vehicles (Liu et al., 2009; Zhang et al., 2015; De Silva et al., 2016; Solgi, Roohi & Kouroshi-Gholampour, 2016) and also contains less nutrients than the soil in natural environments (Li et al., 2013). In fact, *Senecio vulgaris* has been found to be tolerant to lead and cadmium stress (Briggs, 1976; Wei, Zhou & Wang, 2003). Bacteria might help *S. vulgaris* resist heavy metals as well as acquire nitrogen and phosphate in contaminated and oligotrophic environments.

In this study, we collected rhizosphere soil and plant samples of *Senecio vulgaris* populations from four sites in the Shennongjia Forestry District, Hubei Province, China. We tested the following hypotheses: (1) plant compartments and sampling locations determine the diversity of the rhizospheric and endophytic bacterial communities associated with *S. vulgaris* plants; and (2) endophytic bacterial communities from different sites share core operational taxonomic units (OTUs). To test these hypotheses, we examined bacterial communities in the rhizosphere and leaf and root endospheres of *S. vulgaris* populations using Illumina amplicon sequencing and analysis of the bacterial 16S rRNA genes. We also discuss the functions of some top core endophytic bacterial OTUs of *S. vulgaris* plants based on previous studies.

## Materials and Methods

### Sample collection and processing

**Figure 1:**
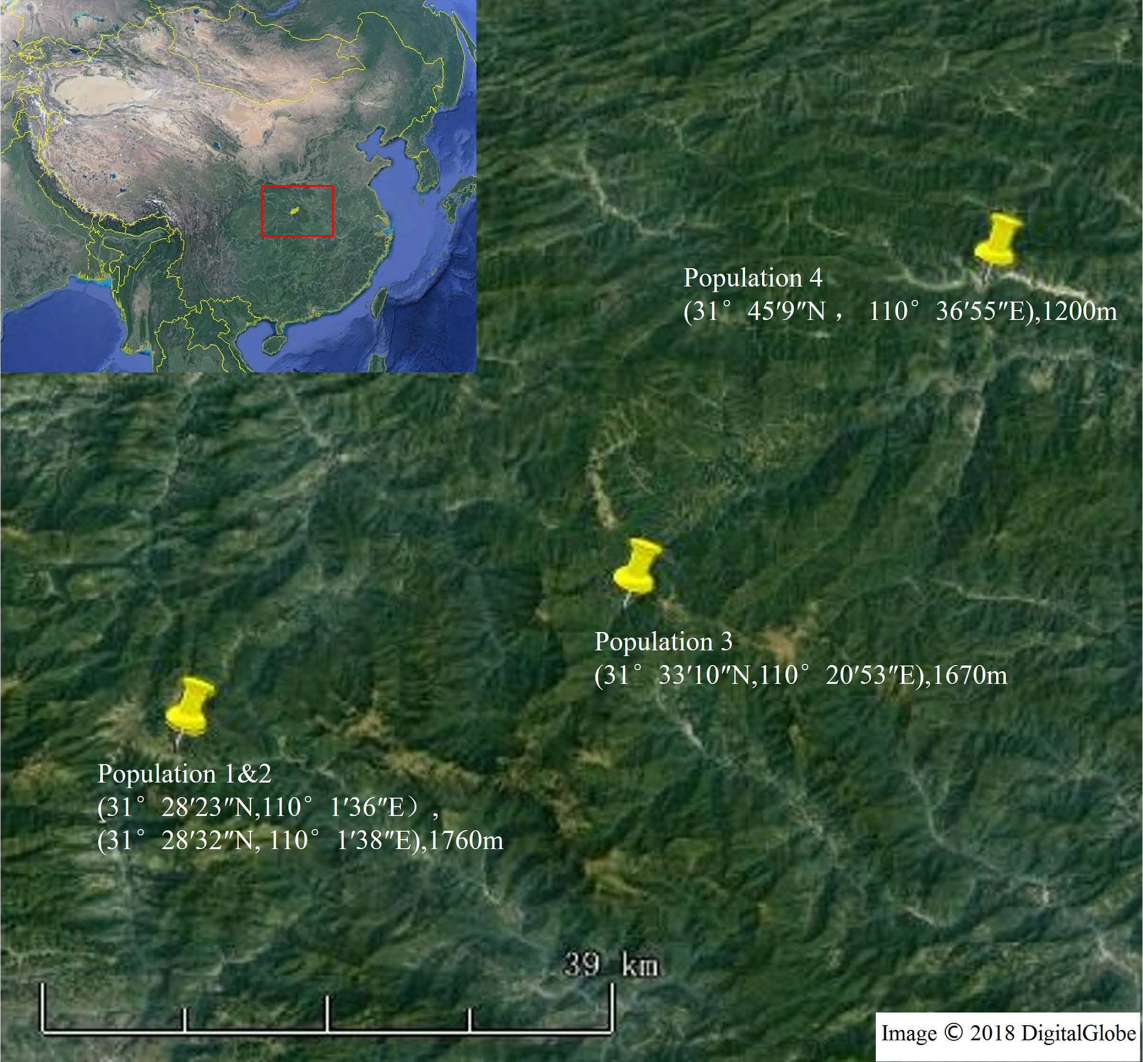
Four sampling locations in Shennongjia, Hubei Province, China. Map data: Google Earth, DigitalGlobe.

We collected rhizosphere soil, leaf and root samples of *S. vulgaris* plants from four locations selected at random to represent the different habitat types of *S. vulgaris* (arable land, wasteland, and roadsides) in our study area. Some of the sampled plants grew in cracks between concrete blocks, rocks, and bricks. All samples were collected in April of 2016 in Shennongjia Forestry District, Hubei Province (Fig. 1). In Shennongjia, the annual temperature is 12 °C, annual precipitation ranges from 800 to 2,500 mm, and the elevation ranges from 398 to 3,105 m above sea level. In March and April 2016, the daily minimum temperature in Shennongjia is often below 10 °C (Fig. S1). The vertical vegetation spectrum along the sampling sites consisted of mixed deciduous and evergreen broad-leaved forest (1,000–1,700 m) and deciduous forest (1,600–2,100 m).

Five squares were established in three locations, and four squares were established in the fourth location. Thus, 19 squares were used in our experiment in total. The dimensions of the squares were 1 × 1 m. A pool of at least three individual plants from one square of a given location was used as one sample. From each square, we collected one rhizosphere, one root, and one leaf endosphere sample. The squares in each location were used as replicates. A total of 57 samples were analyzed.

At each sampling point, the distance between each square was greater than five m. In each square, more than three healthy *S. vulgaris* plants were gently pulled out of the ground, and the soil around the roots was shaken off. Rhizosphere soil was extracted from the root-adhering soil, which was the soil that remained attached to the roots after shaking (Guyonnet et al., 2017). We then placed these plants into a sterile plastic bag, which was subsequently sealed and stored at 4 °C until arrival at the laboratory, at which time the samples were treated immediately.

We placed the roots with the root-adhering soil from one square into a 50 mL centrifuge tube, after which they were rinsed with sterile water and centrifuged for 5 min at 2,000×*g*. The supernatant was then discarded, while the soil that remained in the tube was stored at −80 °C and used as rhizosphere soil for the DNA extraction.

We randomly selected healthy and undamaged leaves and roots, and then attempted to remove the microorganisms from the leaf and root surfaces using the following steps: the samples were washed with ultrapure water, soaked and oscillated for 1 min with 70% alcohol, and then washed for 1 min (leaves) or 5 min (roots) with 1% sodium hypochlorite solution, and then finally rinsed four times with sterile water (Richter-Heitmann et al., 2016). Next, 0.1 mL of the final wash was spread on trypticase soy agar plates to check for contamination (Siciliano & Germida, 1999).

Approximately two g of plant tissue was macerated with a sterile pestle and mortar with liquid nitrogen, and 0.25–0.3 g of finely ground material of soil or plant tissue was used for DNA extraction. We extracted DNA using the MOBIO Power Soil DNA Isolation Kit (MO-BIO, Carlsbad, CA, USA) according to the manufacturer’s instructions.

### PCR amplification and next-generation sequencing

We used 16S rRNA gene amplicons to determine the diversity of the bacterial communities in each of the samples. For polymerase chain reaction (PCR), we used primers 799F (5′-AACMGGATTAGATACCCKG-3′) and 1193R (5′-ACGTCATCCCCACCTTCC-3′), which were designed to specifically amplify the V5, V6, and V7 hypervariable regions of the 16S rRNA gene of bacterial DNA while excluding amplification of chloroplastic DNA from the plants, as suggested in some previous studies (Chelius & Triplett, 2001; Bulgarelli et al., 2012; Bodenhausen, Horton & Bergelson, 2013; Beckers et al., 2016).

Polymerase chain reaction was conducted in 30 μL reactions with Phusion® High-Fidelity PCR Master Mix (New England Biolabs, Ipswich, MA, USA) containing 0.2 μM of forward and reverse primers and about 10 ng template DNA. The thermal cycling consisted of initial denaturation at 98 °C for 1 min, followed by 30 cycles of denaturation at 98 °C for 10 s, annealing at 50 °C for 30 s, and elongation at 72 °C for 30 s, and then a final extension at 72 °C for 5 min.

The size of PCR products was checked on 2% agarose gels. Samples with a bright main band between 400 and 450 bp were selected for further experiments. PCR products were purified with a Qiagen Gel Extraction Kit (Qiagen, Düsseldorf, Germany), and sequencing libraries were generated using a TruSeq® DNA PCR-Free Sample Preparation Kit (Illumina, San Diego, CA, USA) following the manufacturer’s recommendations. Purified samples were quantified with a Qubit Fluorometer (Invitrogen, Waltham, MA, USA), and an equivalent quantity of DNA for each sample was pooled together. In addition, index codes were added to the libraries.

The library quality was assessed using a Qubit Fluorometer (Invitrogen, Waltham, MA, USA) and Bioanalyzer 2100 system (Agilent, Santa Clara, CA, USA). Finally, the library was sequenced on an Illumina HiSeq2500 platform and 250 bp paired-end reads were generated. Sequencing was conducted at Novogene Bioinformatics Technology Co., Ltd (Beijing, China).

### Sequence data treatment

Paired-end reads were assigned to samples based on their unique barcode, truncated by cutting off the barcode and primer sequence, and then merged using FLASH (V1.2.7, http://ccb.jhu.edu/software/FLASH/). Quality filtering of the raw tags was performed under specific filtering conditions to obtain high-quality clean tags according to the QIIME (V1.7.0, http://qiime.org/index.html) quality-controlled process. The tags were compared with those in a reference database (Gold Database, http://drive5.com/uchime/uchime_download.html) using the UCHIME algorithm (http://www.drive5.com/usearch/manual/uchime_algo.html) to detect chimeric sequences, which were removed to yield the effective tags.

Sequence analyses were performed with Uparse software (Uparse v7.0.1001, http://drive5.com/uparse/), and sequences with ≥97% similarity were assigned to the same OTU. Representative sequences for each OTU were then screened for further annotation. For each representative sequence, the GreenGene Database (http://greengenes.lbl.gov) was employed based on the RDP classifier (Version 2.2, http://sourceforge.net/projects/rdp-classifier/) algorithm to annotate taxonomic information.

Of the 3,046,898 high-quality reads that were originally obtained, 2,620,319 sequences were used for further analysis after removing the OTUs that were not classified as bacterial or that matched chloroplasts, mitochondria, or Viridiplantae. The average length of the sequences was 375 nt. Fewer sequences were obtained from the leaf samples than from the root and soil samples. An average of 42% reads from the leaf samples were plastid or mitochondrial. After removal of plastid and mitochondrial contaminants, the leaf samples still had an average of 36,040 usable reads (Table S1). In total, 2,617,582 reads were annotated to 34 bacterial phyla, 2,445,328 reads were annotated to 275 families, and 721,155 reads annotated to 246 species (Table S2).

The abundance of the OTUs was normalized using a standard sequence number corresponding to the sample with the lowest number of sequences. From the raw reads, we produced 4,918 OTUs. After normalization, ca. 500 OTUs were eliminated. Most of the eliminated OTUs were from soil samples and had fewer than 10 reads across all of the samples. Ultimately, 4,369 bacterial OTUs were used in the downstream analyses.

### Selection of core bacterial OTUs in the endosphere

The core OTUs were manually selected based on the average relative abundance and the relative frequency (rf) of each OTU per compartment. The rf of each OTU was calculated using the following formula: rf = number of samples in which a certain OTU was present/total number of samples. We first ranked the OTUs from highest relative abundance to lowest and then selected a certain number of top OTUs that collectively comprised about 80% of the total abundance of the bacterial community. This is similar to the Pareto concept (the 80–20 rule) applied in microbiological community analysis as suggested by Werner et al. (2011). After their identification, we plotted the average relative abundance and frequency of the core OTUs across each sample type.

### Statistical analyses

Analyses of alpha and beta diversity were performed based on the output normalized data. We calculated the Shannon’s diversity index (H′) using the “diversity” function in the vegan package (Dixon, 2003), while Venn diagrams were plotted with the “venn.diagram”function of the VennDiagram package (Chen, 2016). Differences in the bacterial alpha diversities between compartments and locations were compared by multiple comparisons of Shannon index (H′) means between different groups of samples accomplished using the function “glht” in the multcomp package, as this function offers a robust procedure for comparing multiple means under heteroscedasticity (Herberich, Sikorski & Hothorn, 2010). Tukey post hoc tests were used for multiple comparisons, and the single-step method was used to adjust the *P*-values (Table S3).

The Welch’s *t*-test is similar to the Student’s *t-*test and can be applied when the two compared groups have unequal variances. The abundance data of the top five phyla and top 10 families had unequal variances between the different plant compartments. Hence, to determine whether the abundances of the top five phyla and top 10 families differed significantly between the plant compartments, we conducted Welch’s *t*-tests using STAMP software (Parks et al., 2014).

Bray–Curtis distances between the samples were calculated using the function “vegdist” within the vegan package. Nonmetric multidimensional scaling (NMDS) was performed using the vegan packages. Permutational multivariate analysis (PERMANOVA), corroborated by the NMDS plots and using the Bray–Curtis distance, was performed with the function “adonis” in the vegan package as described in Desgarennes et al. (2014).

With the exception of the Welch’s *t*-tests, all analyses were conducted using R (R Core Team, 2015).

## Results

### Alpha-diversity of the bacterial communities

**Figure 2:**
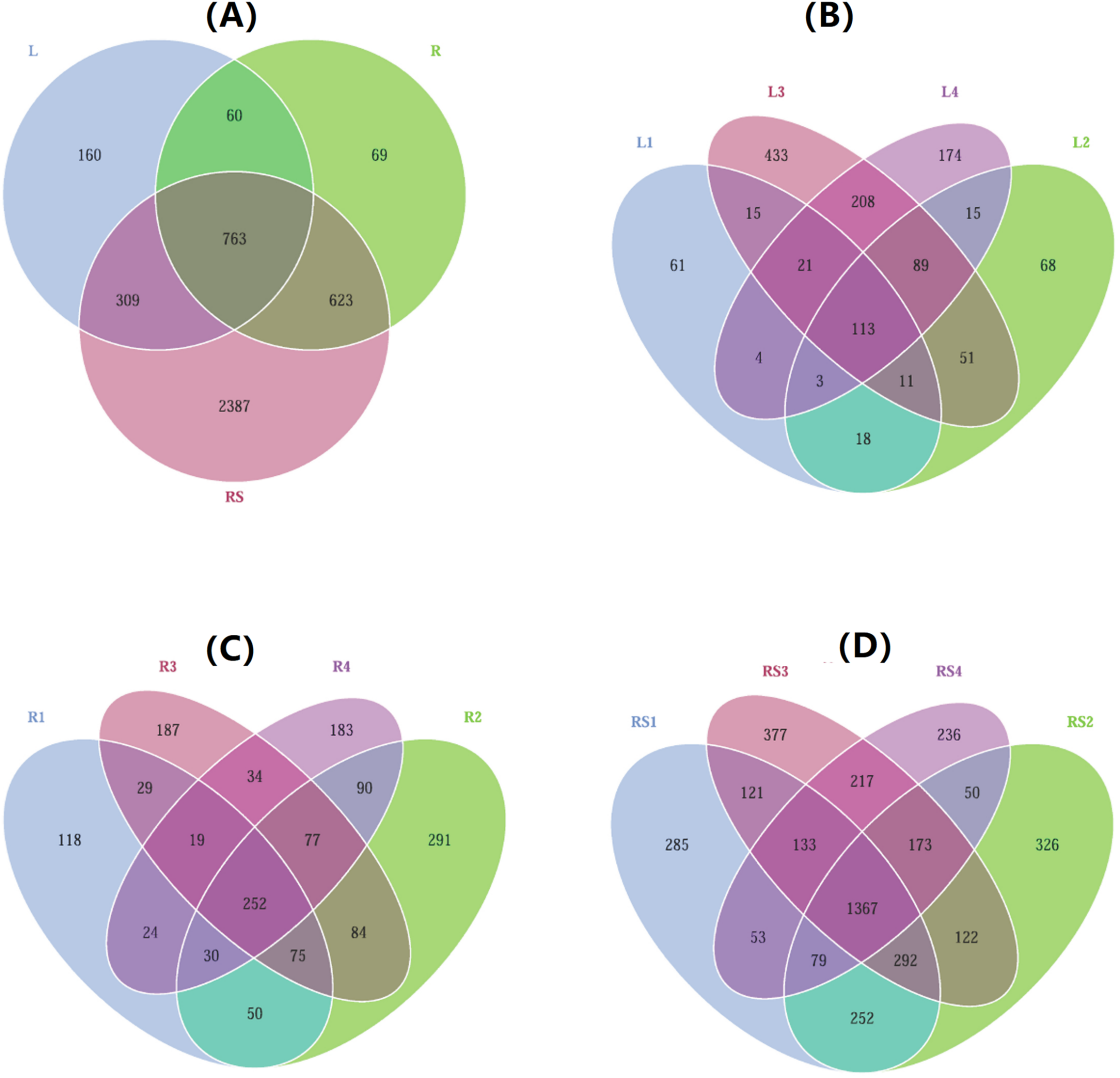
Venn diagrams of shared OTUs (number of OTUs) across three compartments of *S. vulgaris* plants and four sampling locations. (A) Shared OTUs across three compartments of *S. vulgaris*. (B) Shared OTUs of the leaf endosphere across four locations. (C) Shared OTUs of the root endosphere across four locations. (D) Shared OTUs of the rhizospheres across four locations. Letters in the figure: L = leaf endosphere, R = root endosphere, RS = rhizosphere; 1–4 represents the four sampling locations. The diagrams were calculated on a rarefied dataset.

The majority of the bacterial OTUs identified in the leaf and root endosphere were also present in the rhizosphere. Moreover, 289 OTUs were detected solely in the endophytic bacterial communities, which was a considerably small number and only 6.6% of all the identified OTUs. Additionally, only 160 and 69 OTUs were exclusively observed in the leaves and roots, representing 12.4% and 4.6% of the leaf and root communities, respectively (Fig. 2A). The percentage of OTUs shared between the locations was 33%, 17%, and 9% for the rhizosphere, root, and leaf samples, respectively (Figs. 2B–2D).

**Figure 3:**
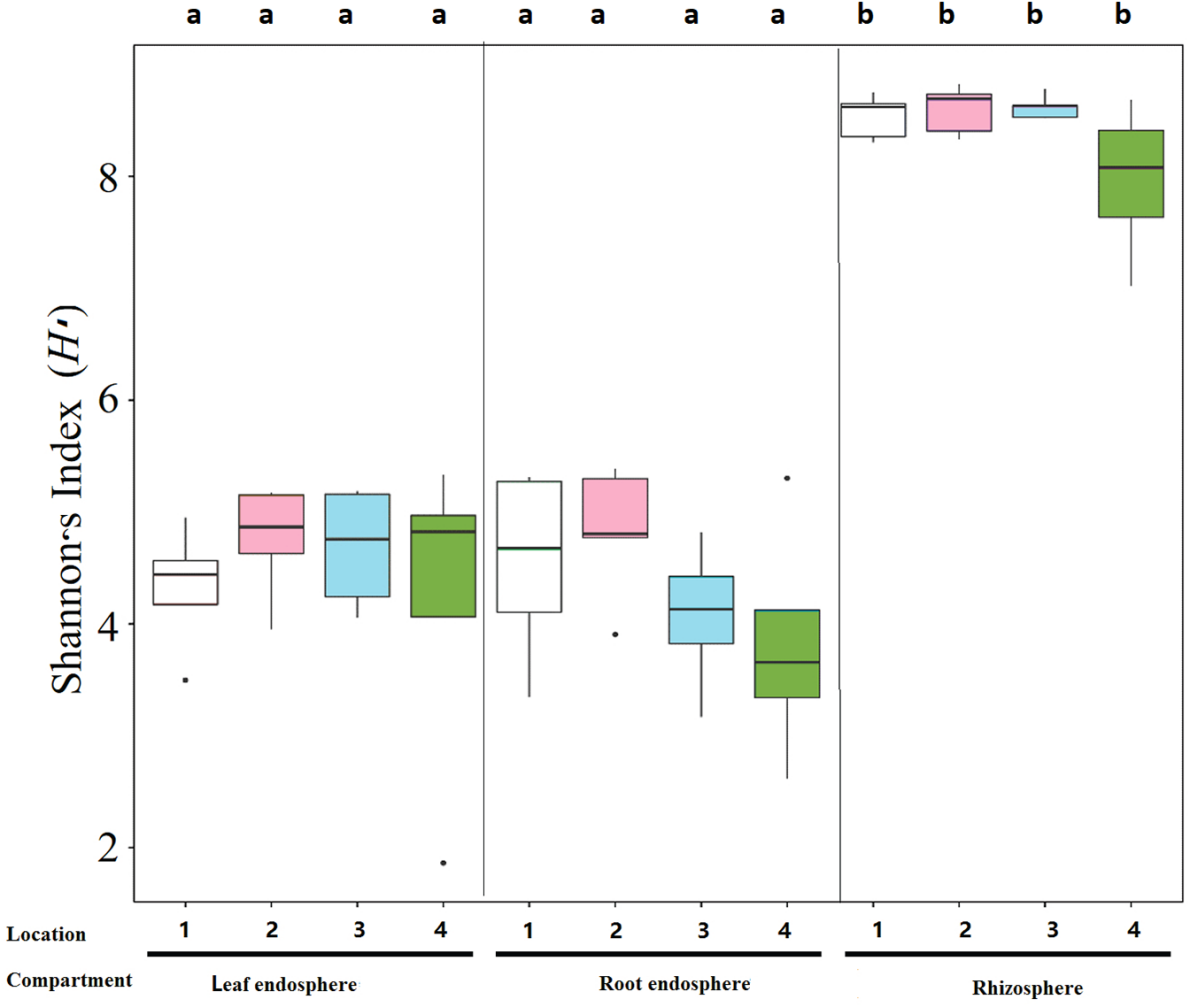
Boxplots of the estimated Shannon index (H′) in the bacterial communities of each compartment of *S. vulgaris* plants and sampling locations. Letters on the top of the boxplots show the results of multiple comparisons of the Shannon index between different groups of samples, and further details of these comparisons are provided in Table S3.

The levels of microbial diversity differed significantly among the compartments. Alpha diversity measured by the Shannon index (H′) was affected by compartments, but not by locations. Specifically, H′ decreased significantly from the soil to the root or leaf endospheres (Fig. 3; Table S3).

### Bacterial community composition

**Figure 4:**
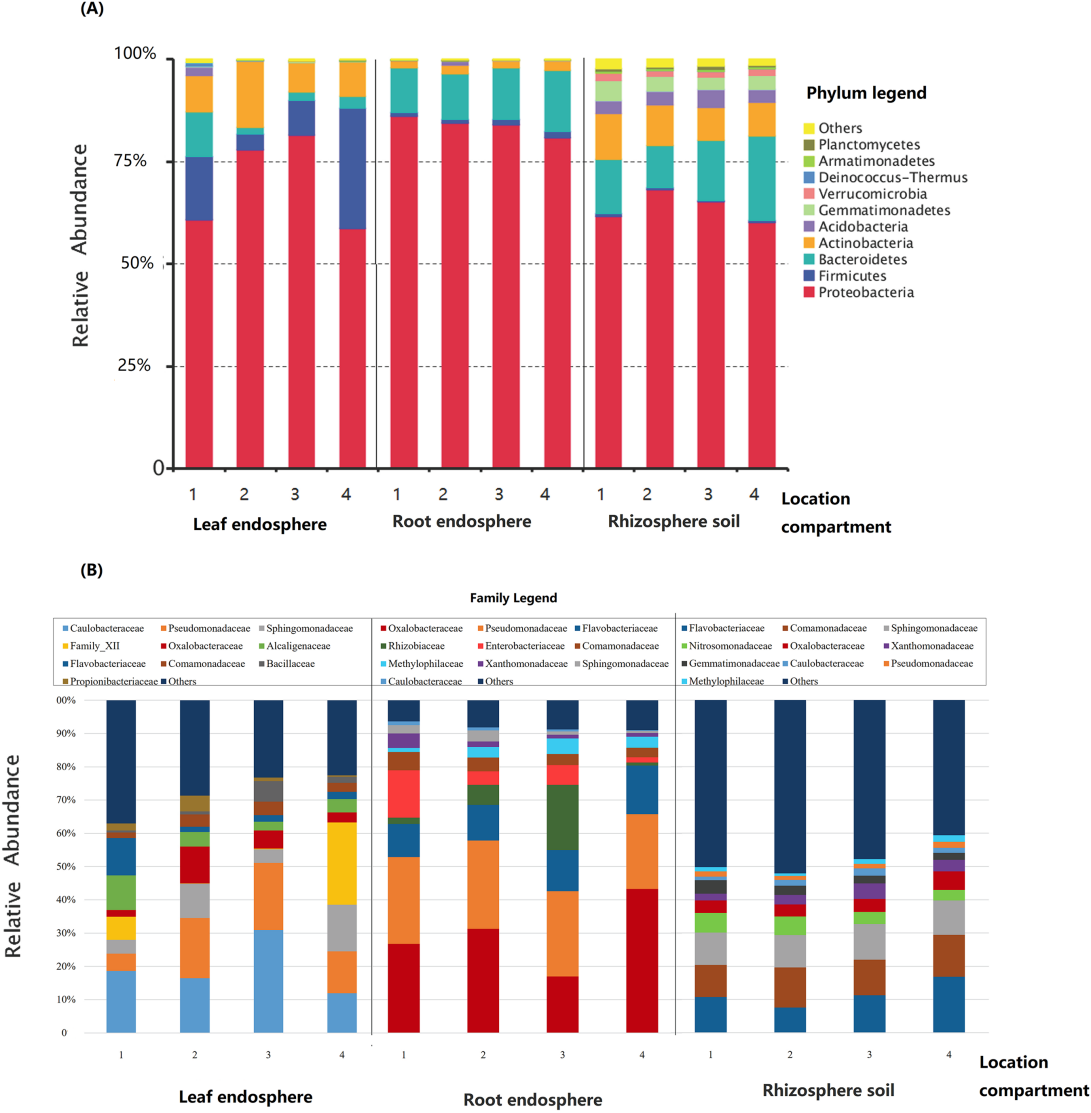
Relative abundance of the top 10 phyla and families in the bacterial communities associated with each compartment of *Senecio vulgaris* plants and sampling locations. (A) The top 10 phyla. (B) The top 10 families.

**Figure 5:**
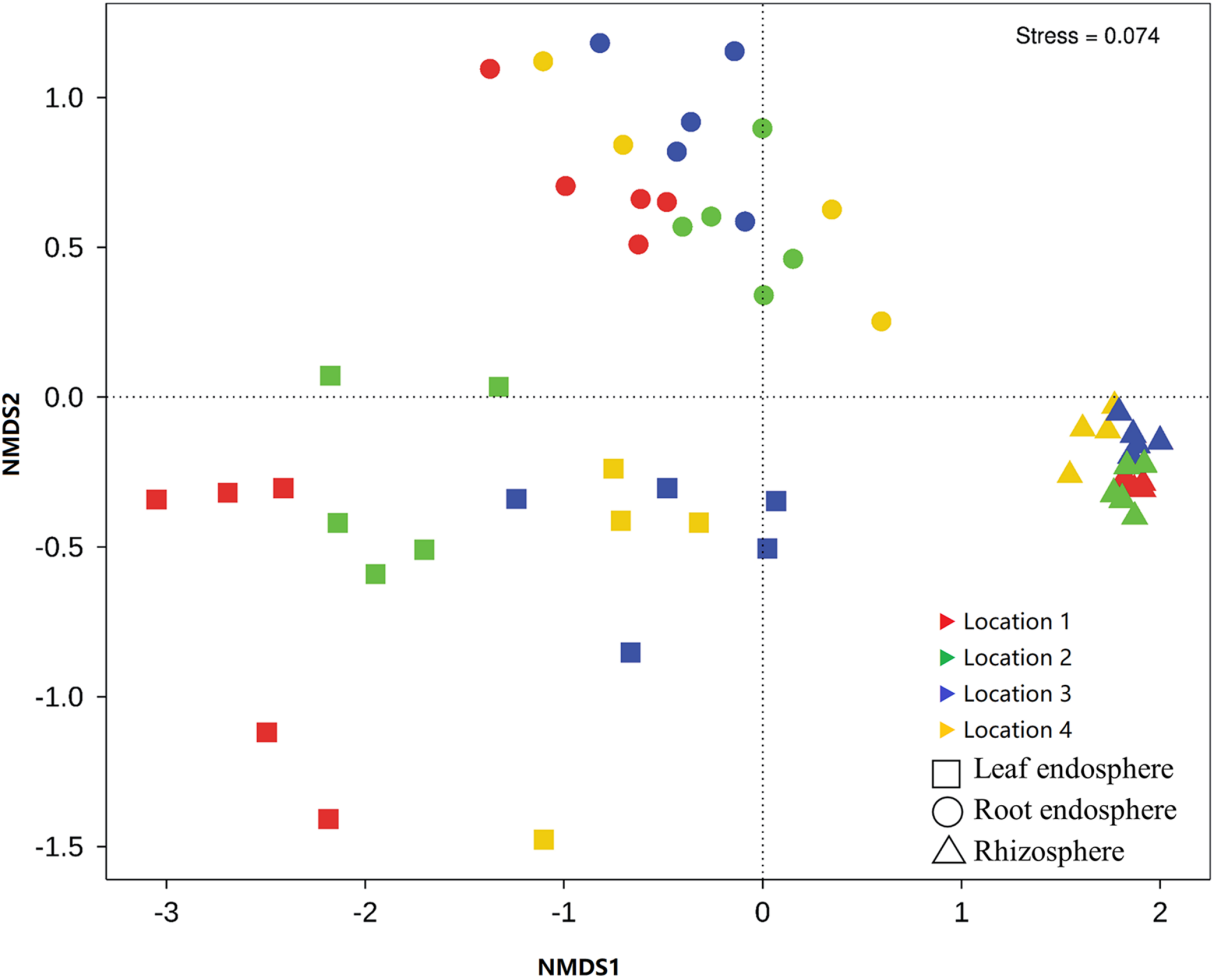
Nonmetric multidimensional scaling (NMDS) plots for Bray–Curtis distances of the bacterial communities associated with each compartment of *Senecio vulgaris* plants and sampling locations.

Across all samples, we detected a total of 34 distinct bacterial phyla, among which the top 10 phyla comprised an average of >98% bacterial abundance in all samples, and the top five (Proteobacteria, Firmicutes, Bacteroidetes, Actinobacteria, and Acidobacteria) comprised an average of >90% of the bacterial abundance (Fig. 4A). Samples from the different compartments differed from one another in relation to the relative abundance of the five dominant phyla: rhizosphere bacterial communities were enriched for Acidobacteria; root endosphere samples had a high abundance of Proteobacteria; and leaf endosphere samples had the highest abundance of Firmicutes (Fig. 4A). The bacterial community composition differed between compartments at the family level. Rhizosphere bacterial communities had higher abundances of Flavobacteriaceae and Sphingomonadaceae, while Oxalobacteraceae and Pseudomonadaceae were most abundant in the root endosphere and Caulobacteraceae were enriched in the leaf endosphere (Fig. 4B). The significance of these differences was confirmed by the Welch’s *t*-tests (Figs. S2 and S3). Bacterial community composition also differed substantially between the compartments and locations in the relative abundance profiles at the OTU level (Fig. 5; Table 1).

**Table 1:** Results of the multiple comparisons of the permutational ANOVA(PERMANOVA) tests on bacterial communities in the leaf endosphere, root endosphere, and rhizosphere of *Senecio vulgaris* plants from different locations.

Comparison pair^1^	DF	*F*	*R*^2^	*P*
L1–L2	1(8)	2.586	0.244	0.001**
L1–L3	1(8)	3.310	0.293	0.006**
L1–L4	1(7)	1.936	0.217	0.025*
L2–L3	1(8)	1.708	0.176	0.045*
L2–L4	1(7)	1.570	0.183	0.014*
L4–L3	1(7)	1.812	0.206	0.019*
R1–R2	1(8)	2.026	0.202	0.105 ns
R1–R3	1(8)	2.170	0.213	0.029*
R1–R4	1(7)	2.520	0.265	0.04*
R2–R3	1(8)	2.101	0.208	0.039*
R2–R4	1(7)	2.038	0.225	0.052 ns
R3–R4	1(7)	1.089	0.135	0.302 ns
RS1–RS2	1(8)	2.359	0.228	0.041*
RS1–RS3	1(8)	4.596	0.365	0.006**
RS1–RS4	1(7)	3.426	0.329	0.001**
RS2–RS3	1(8)	3.222	0.287	0.012*
RS2–RS4	1(7)	3.044	0.303	0.009**
RS3–RS4	1(7)	1.433	0.170	0.08 ns

**Notes:**

ns, not significant.

Significance code:

* *P* < 0.05,

** *P* < 0.01.

1 Explanation for the sample groups: L = leaf endosphere, R = root endosphere, RS = rhizosphere; 1–4 represents the four locations for sampling.

### Core bacterial OTUs in the root and leaf endospheres

**Figure 6:**
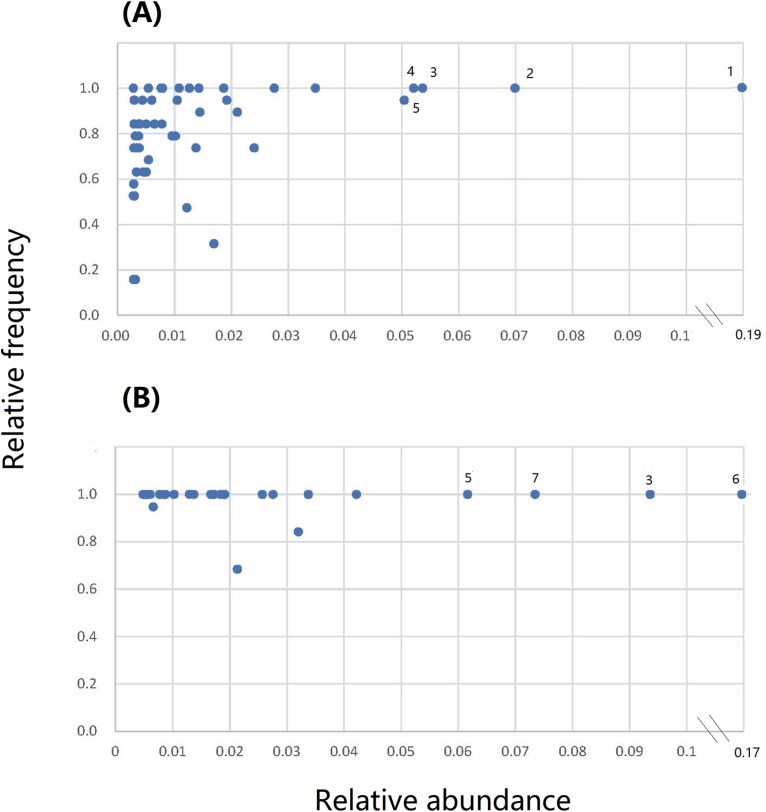
The relative frequency vs relative abundance of core bacterial operational taxonomic units (OTUs) in the root and leaf endospheres of *S. vulgaris* plants. (A) The root endospheres. (B) The leaf endospheres. OTUs: 1 = *Brevundimonas diminuta*, 2 = *Exiguobacterium sibiricum*, 3 = *Pseudomonas* spp., 4 = an undefined species from Alcaligenaceae, 5 = *Pseudomonas viridiflava*, 6 = an undefined species from Oxalobacteraceae, and 7 = *Duganella* spp. The relative frequency (rf) of an OTU was calculated using the following formula: rf = number of samples in which a certain OTU was present/number of all samples.

From the 1,284 OTUs in the leaf endosphere, we identified 36 OTUs with >0.70 rf as core OTUs that collectively comprised more than 70% of the leaf endophytic bacterial communities (Table S4). The endosphere bacterial communities were dominated by a few bacterial phyla or orders, including Alpha-, Beta-, and Gammaproteobacteria, Actinobacteria, Firmicutes (Bacilli), and Bacteroidetes (Flavobacteria, Table 2).The top five OTUs in the leaf endosphere were *Brevundimonas diminuta* (Alphaproteobacteria), *Exiguobacterium sibiricum* (Bacilli), *Pseudomonas* sp. (OTU7, Gammaproteobacteria), OTU6 (Alcaligenaceae, Betaproteobacteria), and *Pseudomonas viridiflava* (Gammaproteobacteria, Fig. 6A; Table S4).

**Table 2:** The bacterial taxa dominate in the endosphere of *Senecio vulgaris*.

Phylum	Class	Order	Family	Genus
In leaves and roots				
Bacteroidetes	Flavobacteriia	Flavobacteriales	Flavobacteriaceae	Chryseobacterium
				Flavobacterium
Proteobacteria	Alphaproteobacteria	Sphingomonadales	Sphingomonadaceae	Sphingomonas
	Gammaproteobacteria	Enterobacteriales	Enterobacteriaceae	/
		Pseudomonadales	Pseudomonadaceae	Pseudomonas
Only in leaves				
Actinobacteria	Actinobacteria	Corynebacteriales	Corynebacteriaceae	Corynebacterium
			Mycobacteriaceae	Mycobacterium
		Micrococcales	Brevibacteriaceae	Brevibacterium
			Micrococcaceae	Kocuria
		Propionibacteriales	Propionibacteriaceae	Propionibacterium
Firmicutes	Bacilli	Bacillales	Bacillaceae	Bacillus
			Family_XII	Exiguobacterium
			Staphylococcaceae	Staphylococcus
Proteobacteria	Alphaproteobacteria	Caulobacterales	Caulobacteraceae	Brevundimonas
		Rhizobiales	Bradyrhizobiaceae	Bosea
				Bradyrhizobium
			Rhizobiaceae	Ensifer
	Betaproteobacteria	Burkholderiales	Alcaligenaceae	/
			Comamonadaceae	Pelomonas
				Variovorax
			Oxalobacteraceae	/
				Duganella
				Massilia
	Gammaproteobacteria	Pseudomonadales	Moraxellaceae	Acinetobacter
Only in roots				
Proteobacteria	Alphaproteobacteria	Rhizobiales	Rhizobiaceae	Rhizobium
		Sphingomonadales	Sphingomonadaceae	Sphingobium
	Betaproteobacteria	Burkholderiales	Comamonadaceae	Acidovorax
		Methylophilales	Methylophilaceae	Methylophilus
				Methylotenera
	Gammaproteobacteria	Xanthomonadales	Xanthomonadaceae	Stenotrophomonas

**Notes:**

This table summarized the taxa information of the core OTUs in the endosphere of *S. vulgaris* plants, and the abundance data of the core OTUs are provided in Tables S4 and S5.

/ = unidentified taxa.

Similarly, from the 1,543 OTUs, we identified 30 OTUs as core root endophytic bacteria. The four most abundant were: OTU3 (Oxalobacteraceae, Betaproteobacteria), *Pseudomonas* sp. (OTU7), *P. viridiflava*, and *Duganella* sp. (OTU15, Betaproteobacteria, Fig. 6B; Table S5). With the exception of three OTUs, the core root endophytic bacteria were present in all of the root samples. These OTUs collectively comprised more than 70% of the root endophytic bacterial communities (Table S5).

## Discussion

### Differences between the plant compartments and sampling locations

We determined that the bacterial communities associated with *Senecio vulgaris* were primarily influenced by plant compartments, as the alpha diversity was significantly decreased in the root and leaf endospheres compared with the rhizosphere soil (Fig. 3; Table S3). These findings were consistent with observations from many other plants, such as *Agave* species (Coleman-Derr et al., 2015), rice (Edwards et al., 2015), and poplar (Beckers et al., 2016). Microbial diversity declines sequentially from the rhizosphere to the root and leaf endospheres, which suggests increasingly stronger competition among microorganisms as the habitat becomes more tightly defined (Müller et al., 2016). However, specific communication may occur in these compartments because of specific plant metabolites (Haichar et al., 2014).

Although most bacteria in the root and leaf endospheres were recruited from the rhizosphere, the bacterial community structures in these three compartments were clearly distinct from one another. Proteobacteria, Actinobacteria, Firmicutes, and Bacteroidetes dominated the rhizosphere and endosphere of *S. vulgaris*. However, the relative abundance of Proteobacteria and Firmicutes increased, while that of Acidobacteria decreased from the rhizosphere to the endosphere. These findings regarding the varying phyla distributions between the plant compartments are consistent with observations from other plants, including rice (Edwards et al., 2015), maize (Liu et al., 2017), grape (Zarraonaindia et al., 2015), agave (Coleman-Derr et al., 2015), *Brassica stricta* (Wagner et al., 2016), *Oxyria digyna*, and *Saxifraga oppositifolia* (Kumar et al., 2017). In combination, these results indicate that there may be certain factors that shape the structure of the rhizo- and endophytic bacterial communities acting in different environments and host species. It is suggested that such factors include root exudates in the soil, the physicochemical properties of the plant cell walls, and the metabolites from active plant cells (Haichar et al., 2008; Bulgarelli et al., 2012; Guyonnet et al., 2017). Furthermore, Bulgarelli et al. (2013) proposed a two-step selection model in which rhizodeposition and convergent host genotype-dependent selection drives the community shift in the rhizosphere and endophyte microbiota differentiation. Obviously, this plant selection process can explain the differentiation between the bacterial microbiota in the endosphere and in the soil.

We also found that the bacterial communities associated with *S. vulgaris* were influenced by sampling locations that were far from one another or in different types of habitats. This type of influence is often the result of differences between climate and soil physiochemical properties between locations (Werner et al., 2011; Coleman-Derr et al., 2015). We were unable to measure the geochemical properties of all the soil samples, and we have here omitted the investigation regarding the influence of these factors on plant microbiota. Recent studies have demonstrated that plant host-specific traits, including broad morphological characteristics (Kembel et al., 2011) and specific genetic pathways and gene products (Horton et al., 2014; Lebeis et al., 2015), can have significant effects on microbiota composition and diversity.

### Core bacterial OTUs in the root and leaf endospheres

When only the profile of the endophytic bacterial OTUs was considered, major differences were observed between the locations (Fig. 5). However, when the abundance of the OTUs was considered, the *S. vulgaris* plants from different locations were found to share similar core OTUs in the leaf and root endospheres. These core OTUs accounted for much less than 20% of the total OTUs but did account for >70% of the abundance of the endophytic bacterial communities. These findings demonstrated that the core taxa of the endophytic bacterium might be consistent across hosts of the same species growing in different locations, as has been observed in *Arabidopsis* (Bulgarelli et al., 2012; Lundberg et al., 2012), grape (Samad et al., 2017), and some other plant species (Kumar et al., 2017).

The dominating phyla or orders, including Alpha-, Beta-, and Gamma-proteobacteria, Actinobacteria, Firmicutes (Bacilli), and Bacteroidetes (Flavobacteria), also tend to be the dominant endophytic bacteria of other plants, as reviewed by Hardoim et al. (2015), Müller et al. (2016), and Finkel et al. (2017). In *S. vulgaris* plants, the core leaf endophytic bacterial OTUs belonged to 19 families, while those in the roots belonged to 10 families (Table 2; Tables S4 and S5). We compared these dominant families to those reported in previous studies and found that the dominant families in the *S. vulgaris* roots substantially overlapped with those reported as the core set present in *Arabidopsis thaliana*, *Salicornia europaea*, and *Helianthus annuus*. Among the leaf endophytic bacteria, *A. thaliana* shared many families with *S. vulgaris*, while *Sequoia sempervirens* and *Sequoiadendron giganteum* shared few with *S. vulgaris* (Table 3). This comparison indicated that although the structure of the endophytic bacteria communities differed between plant species, similarities in the taxa of the plant bacteriome could be observed at the phylum, order, or even the family level.

**Table 3:** Dominant bacterial families in the root and leaf endosphere of *Senecio vulgaris* plants reported as core members in previous studies.

Famliy	Root endosphere								Leaf endosphere		
	*A. thaliana*	Barely	Rice	*Vitis* spp.	*O. digyna*, *S. oppositifolia*	*P. tremula*, *P. alba*	*S. europaea*	*H. annuus*	*A. thaliana*	*P. tremula*, *P. alba*	*S. sempervirens*, *S. giganteum*
Caulobacteraceae^a^^,^^b^	√					√		√	√		
Pseudomonadaceae^a^^,^^b^	√			√	√		√	√	√	√	
Sphingomonadaceae^a^^,^^b^	√					√	√	√	√	√	
Oxalobacteraceae^a^^,^^b^	√	√		√	√	√		√	√	√	
Flavobacteriaceae^a^^,^^b^	√	√		√	√		√	√	√		
Comamonadaceae^a^^,^^b^	√	√	√	√		√	√	√	√		
Rhizobiaceae^a^	√	√	√				√	√	√	√	
Enterobacteriaceae^a^							√	√	√		√
Methylophilaceae^a^	√							√			
Xanthomonadaceae^a^	√						√	√	√		
Alcaligenaceae^b^	√								√	√	
Family_XII^b^											
Bacillaceae^b^	√								√		
Propionibacteriaceae^b^											

**Notes:**

√ corresponds to bacterial families present as core members. *Arabidopsis thaliana*, barely, and rice are based on Müller et al. (2016) and the references therein; *Vitis* spp. are based on Samad et al. (2017); *Oxyria digyna* and *Saxifraga oppositifolia* are based on Kumar et al. (2017); *Populus tremula* and *Populus alba* are based on Beckers et al. (2016); *Salicornia europaea* is based on Zhao et al. (2016); *Helianthus annuus* is based on Leff et al. (2016); *Sequoia sempervirens* and *Sequoiadendron giganteum* are based on Carrell & Frank (2015).

a Dominant family in the root endosphere of *S. vulgaris*.

b Dominant family in the leaf endosphere of *S. vulgaris* (Fig. 4B).

In the leaf and root bacterial communities of *Senecio vulgaris*, there were several dominant genera, namely *Brevundimonas*, *Pseudomonas*, *Exiguobacterium*, *Sphingomonas*, *Flavobacterium*, *Rhizobium*, *Massilia*, and *Duganella*. Among these, *Pseudomonas* and *Rhizobium* have been thoroughly investigated as plant-associated genera. *Pseudomonas* is known to occupy numerous ecological niches, including the rhizospheres and endospheres of many plants. For instance, 21 *Pseudomonas* strains were isolated from the roots of *Populus deltoides* (Jun et al., 2015), and 12 *Pseudomonas* strains showed promising growth-promoting effects when applied to lettuce in the field (Cipriano et al., 2016). *Massilia* and *Duganella* are in the order Burkholderiales, which is well known for its biodegradative capacities and antagonistic properties toward multiple soil-borne fungal pathogens (Benítez & Gardener, 2009; Chebotar et al., 2015). The genus *Flavobacterium* comprises a significant fraction of the endophytic microbiota in a broad range of plant species, indicating a specialized capacity to proliferate in plant environments and suggesting a role in plant function (Kolton et al., 2016).

### Bacterial function prediction

It would be interesting to determine whether the plant-associated bacteria possess plant-growth-promoting traits (PGPTs), such as the ability to fix nitrogen, solubilize phosphate, produce IAA, hydrogen cyanide, siderophore, and 1-aminocyclopropane-1-carboxylate deaminase, or the possession of antifungal activity. Among the PGPTs, the nitrogen cycle mediated by microbes has been shown to be important in alien plant invasion, such as *Fallopia* spp. in Europe (Bardon et al., 2014, 2016) and *Sorghum halepense* in North America (Rout & Chrzanowski, 2009; Rout et al., 2013).

The *S. vulgaris* dominant endophytes *B. diminuta* and *Rhizobium leguminosarum* may be beneficial to host plants. Singh et al. (2016) applied *B. diminuta* to rice and found that it helped reduce arsenic accumulation and produced IAA to obtain soluble phosphate and promote the growth of rice. Moreover, *R. leguminosarum* biovar. Phaseoli isolated from sludge-treated soil was found to form root nodules in white clover (*Trifolium repens*) (Chaudri, Mcgrath & Giller, 1992; Chaudri et al., 1993). Purchase, Miles & Young (1997) found that *R. leguminosarum* was resistant to heavy metals, particularly cadmium, and that it could effectively conduct nitrogen fixation. In addition, Chabot, Antoun & Cescas (1996) showed that *R. leguminosarum* promoted the growth of maize and lettuce via phosphate solubilization.

We also identified some cold-resistant bacteria as core bacterial OTUs in the root and leaf endospheres of *S. vulgaris*. These included *Sphingomonas aerolata*, *Sphingomonas faeni*, *E. sibiricum*, and OTU 3. Isolates of two *Sphingomonas* species (*Sphingomonas aerolata* and *Sphingomonas faeni*) showed psychrotolerant traits (Busse et al., 2003). *E. sibiricum* is one of 14 known *Exiguobacterium* spp. (Vishnivetskaya, Kathariou & Tiedje, 2009). Strains of this species isolated from Siberian permafrost could grow well at low temperature (e.g., 4 °C) and had remarkable tolerance to repeated freeze-thawing cycles (Vishnivetskaya et al., 2007). OTU3 (Oxalobacteraceae), which may have been a member of either the genera *Duganella*, *Rugamonas*, or *Janthinobacterium*, was highly abundant in the root samples (Fig. 6B; Table S5). *Janthinobacterium lividum* was observed in the endosphere of two native perennial plants, *O. digyna* and *Saxifraga oppositifolia*, in three Arcto-Alpine regions (Kumar et al., 2017). *Janthinobacterium* spp. were reported to thrive in extremely cold, dry, and high solar ultraviolet radiation environments and to manifest strong antimicrobial activity (Koo et al., 2016, and references therein). When our plants were collected in April of 2016 in Shennongjia, we found that *Senecio vulgaris* was one of the weeds that emerged in early spring and that the daily minimum temperature was often below 10 °C (Fig. S1). Therefore, it is not surprising that cold-resistant bacteria are present in the endosphere of *Senecio vulgaris* plants in this region, and it is possible that they could facilitate host growth under cold conditions.

In future studies, we could predict the function of plant-associated bacteria using PICRUST, a bioinformatics software package designed to predict metagenome functional content from marker gene (e.g., 16S rRNA) surveys and full genomes (Langille et al., 2013). The establishment of plant-derived culture collections is also important for the assessment of the biological potential of microbial communities. The combination of culture-dependent and -independent techniques is useful to the study of the role of microbiomes in plant invasion.

## Conclusions

We collected rhizosphere soil, root and leaf endosphere samples in four *Senecio vulgaris* populations in a subtropical mountainous area in Central China. The bacterial 16S rRNA gene data obtained from these samples revealed significant structural differences in the bacterial communities associated with this invasive plant species between different plant compartments and sampling locations. However, similar core bacteria were observed from leaf and root endophytic communities, despite a distance of over 100 km and an elevation range of 1,200–1,800 m between the sampling locations. As expected, we observed heavy metal-resistant, phosphate-solubilizing, and nitrogen-fixing bacteria, such as *B. diminuta* and *R. leguminosarum*, in the *S. vulgaris* roots and leaves at relatively high abundance. These bacteria might be involved in plant adaptions to heavy metal contamination and poor soil nutrition. However, the presence of cold-resistant bacteria was unexpected. The presence of these types of bacteria might be important for the adaptation of *S. vulgaris* to harsh environments. Future studies should be conducted to isolate these endophytes in *S. vulgaris* plants and test their function in vitro and in vivo.

## Supplemental Information

10.7717/peerj.6162/supp-1 Supplemental Information 1 Minimum and maximum temperatures in March and April 2016 in Shennongjia. Temperature data were obtained from the local meteorological office.

10.7717/peerj.6162/supp-2 Supplemental Information 2 Differential abundance of the top five phyla in the bacterial communities across different plant compartments of *Senecio vulgaris*. Welch’s *t*-tests using STAMP software were conducted to determine whether the differences in abundance between plant compartments were significant.

10.7717/peerj.6162/supp-3 Supplemental Information 3 Differential abundance of the top 10 families in the bacterial communities across different plant compartments of *Senecio vulgaris*. Welch’s *t*–tests using STAMP software were conducted to determine whether the differences in abundance between plant compartments were significant.

10.7717/peerj.6162/supp-4 Supplemental Information 4 Number of 16S rRNA gene sequences amplified from each sample. ^*^contamination was from chloroplasts and mitochondria in the host plants.

10.7717/peerj.6162/supp-5 Supplemental Information 5 Number of 16S rRNA gene sequences annotated to different levels amplified from each sample.

10.7717/peerj.6162/supp-6 Supplemental Information 6 Results of the simultaneous tests for general linear hypotheses multiple comparisons of Shannon index between different groups of samples. Explanation for groups of samples: L, leaf endosphere; R, root endosphere; RS, rhizosphere; 1–4 represent the four locations for sampling.

10.7717/peerj.6162/supp-7 Supplemental Information 7 Table S4. Core bacterial OTUs in the leaf endosphere of *Senecio vulgaris* plants. /= unidentified taxa.

10.7717/peerj.6162/supp-8 Supplemental Information 8 Table S5. Core bacterial OTUs in the root endosphere of *Senecio vulgaris* plants. /= unidentified taxa.

10.7717/peerj.6162/supp-9 Supplemental Information 9 Sample names. Explanation for names of the samples used in this study.

10.7717/peerj.6162/supp-10 Supplemental Information 10 DNA sequence data of the bacteria OTUs identified from rhizospere and endosphere of Senecio vulgaris plants.

10.7717/peerj.6162/supp-11 Supplemental Information 11 Absolute abundance of each OTU. This dataset shows number of reads annotated to OTUs. The rows are OTUs and the variables are samples. See explanations for them in the dataset “Sample name”.

10.7717/peerj.6162/supp-12 Supplemental Information 12 Reads number of eliminated OTUs.

10.7717/peerj.6162/supp-13 Supplemental Information 13 Relative abundunce of each OTU. This dataset shows abundance percentage of each OTU in each sample. The rows are OTUs and the variables are samples. See explanations for them in the dataset “Sample name”. The OTUs matching chloroplasts, mitochondrial or Viridiplantae were not in this dataset.

10.7717/peerj.6162/supp-14 Supplemental Information 14 Alpha diversity indexes for each sample.

10.7717/peerj.6162/supp-15 Supplemental Information 15 Relative abundance of the core bacterial OTUs in leaf endosphere of *Senecio vulgaris* plants.

10.7717/peerj.6162/supp-16 Supplemental Information 16 Relative abundance of the core bacterial OTUs in root endosphere of *Senecio vulgaris* plants.

## Additional Information and Declarations

### DNA Deposition

The following information was supplied regarding the deposition of DNA sequences: The sequence data is available in GenBank: https://www.ncbi.nlm.nih.gov/nuccore/?term=MG274323:MG279042[accn]. The data is also available as a Supplemental File.
