# Supplementary figures and images for: Diversity analysis of the rhizospheric and endophytic bacterial communities of Senecio vulgaris L. (Asteraceae) in an invasive range
Source: PeerJ. 2019 Jan 7;6:e6162. doi: 10.7717/peerj.6162 (PMC6327885; doi:10.7717/peerj.6162)

**(a) March**

Daily temperature (°C)

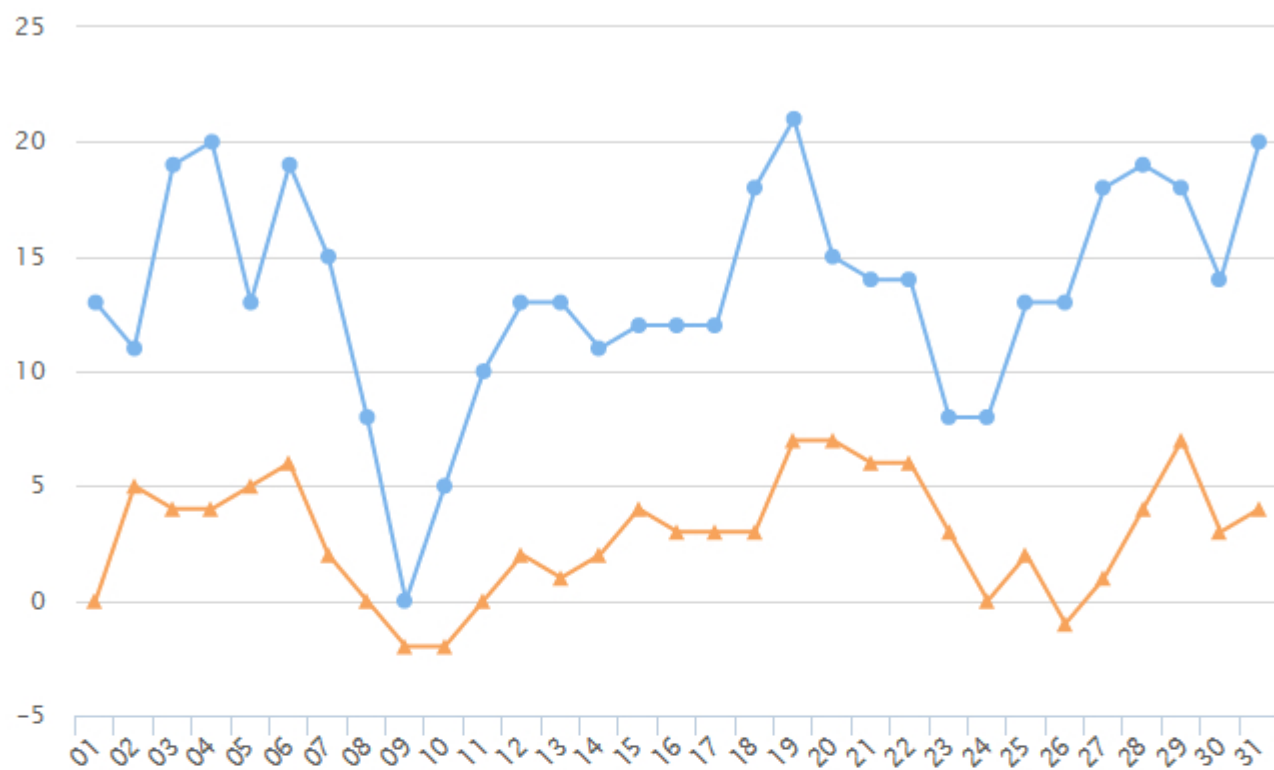

**(b) April**

DATE

Daily temperature (°C)

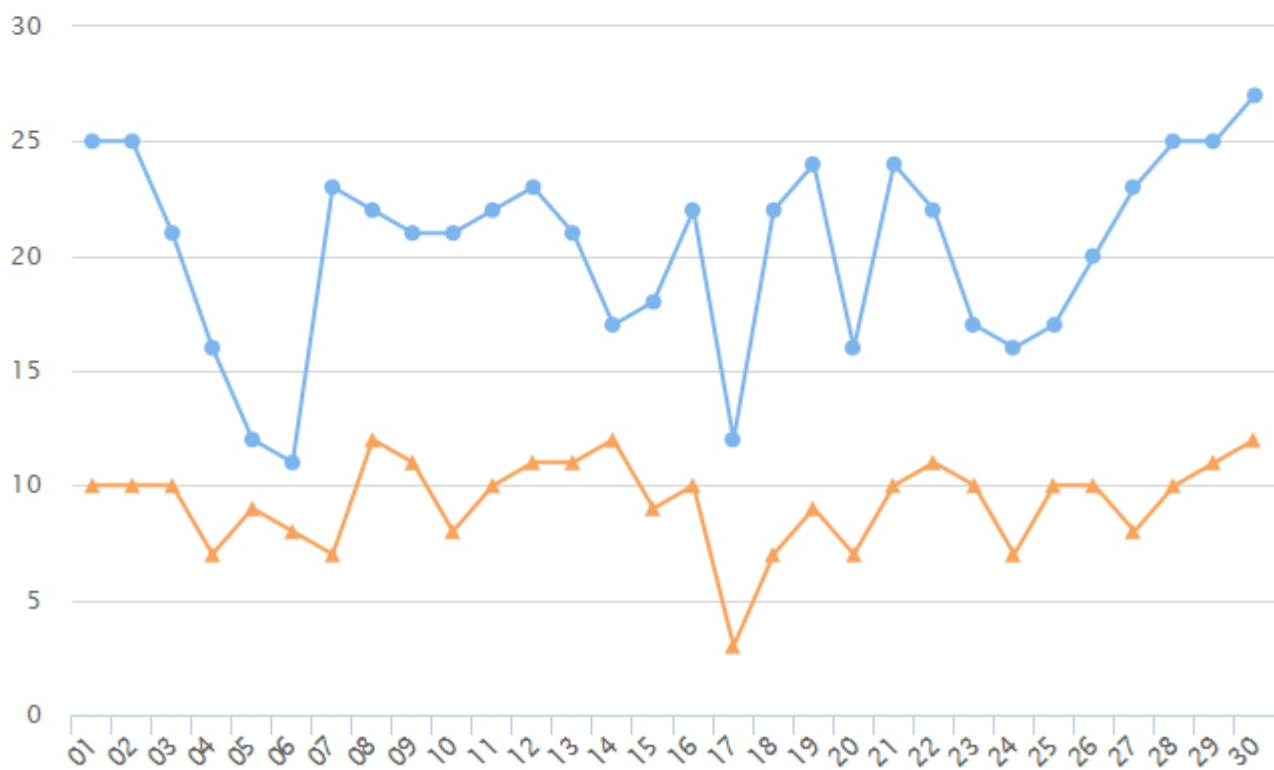

DATE

Supplement: Supplemental Information 1 — Temperature data were obtained from the local meteorological office. [file peerj-07-6162-s001.pdf]

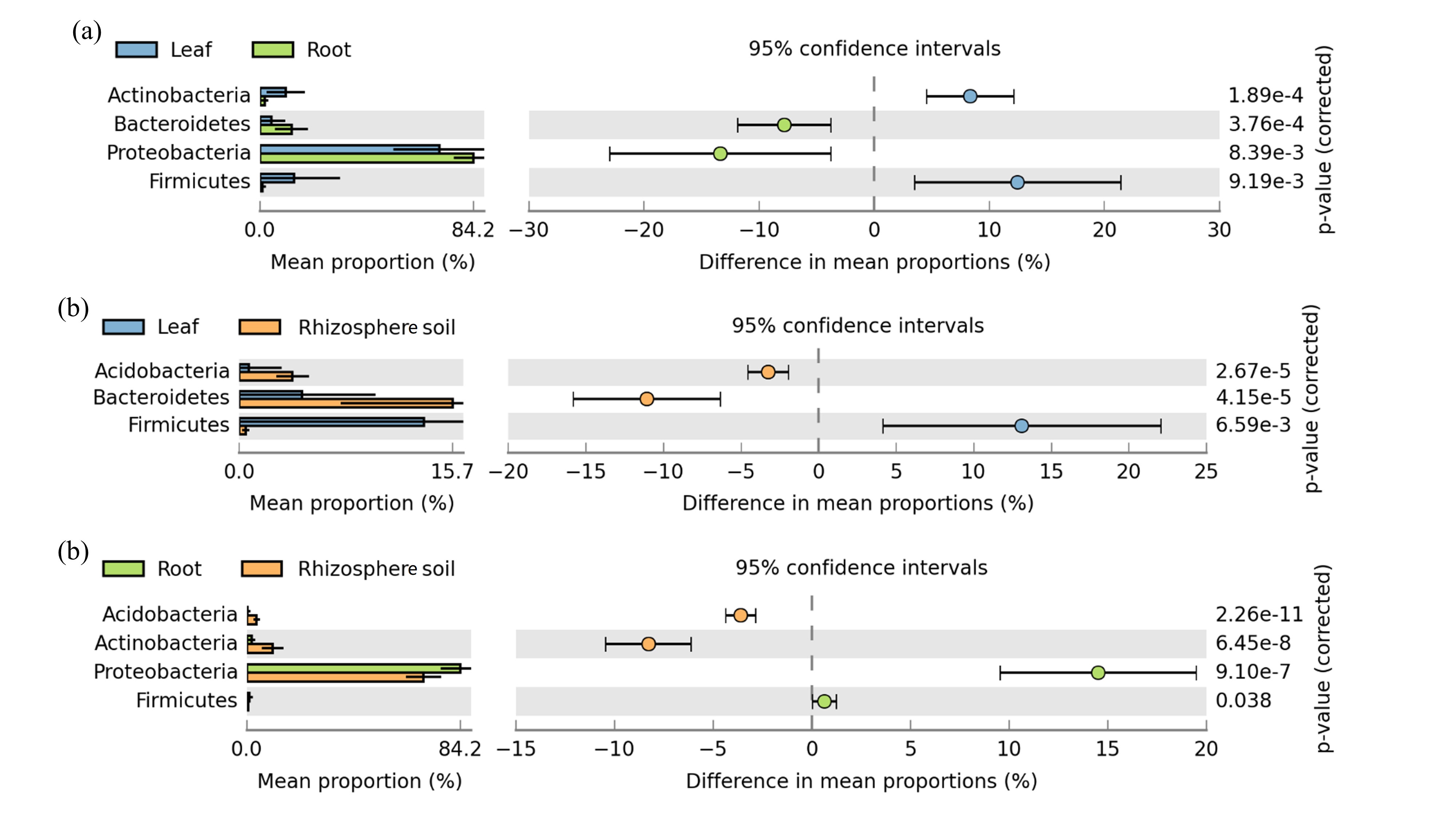

Supplement: Supplemental Information 2 — Welch’s t-tests using STAMP software were conducted to determine whether the differences in abundance between plant compartments were significant. [file peerj-07-6162-s002.jpg]

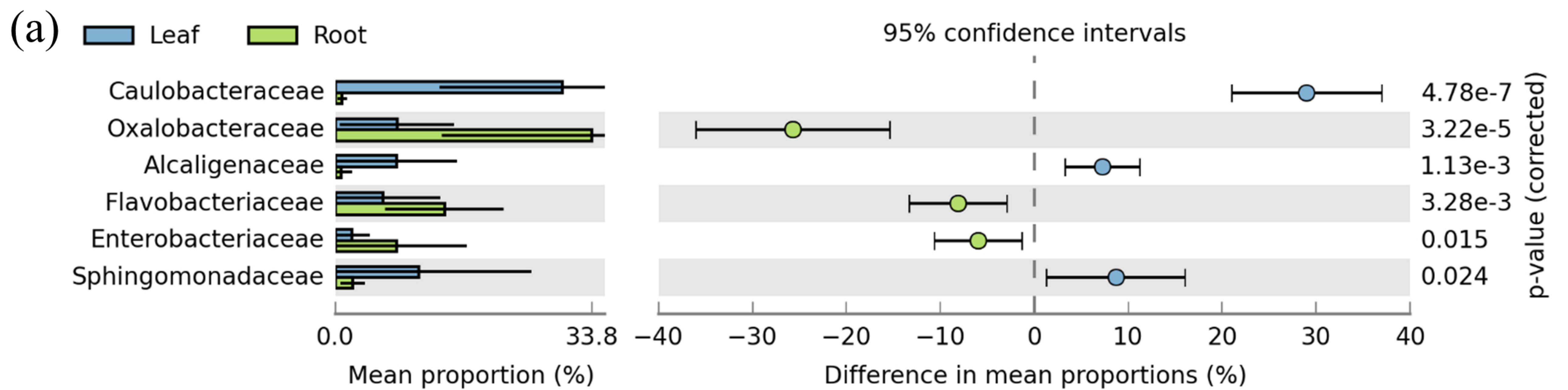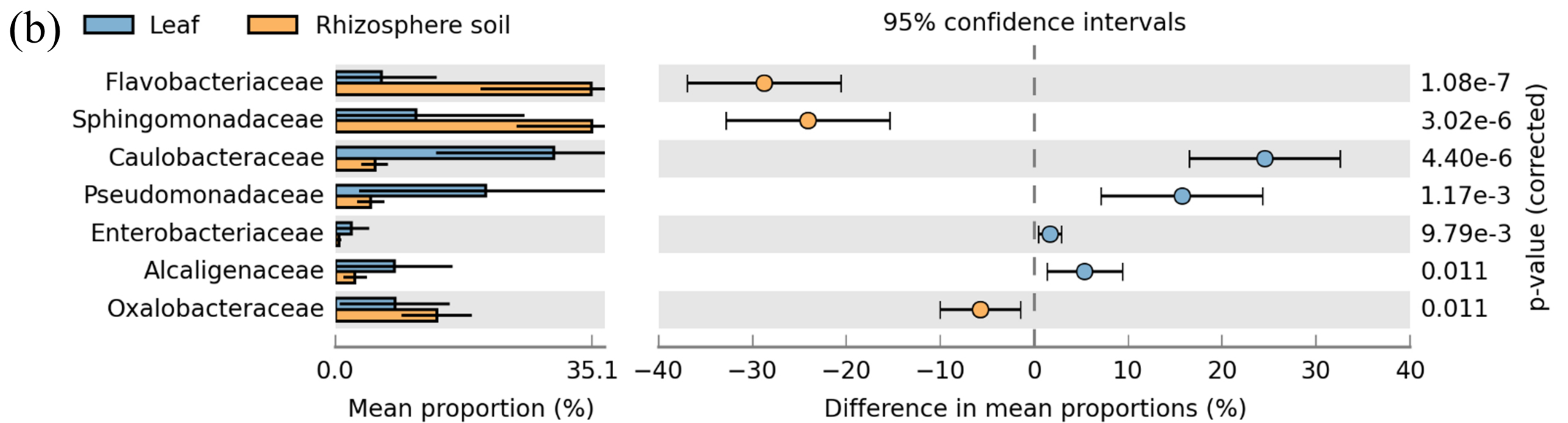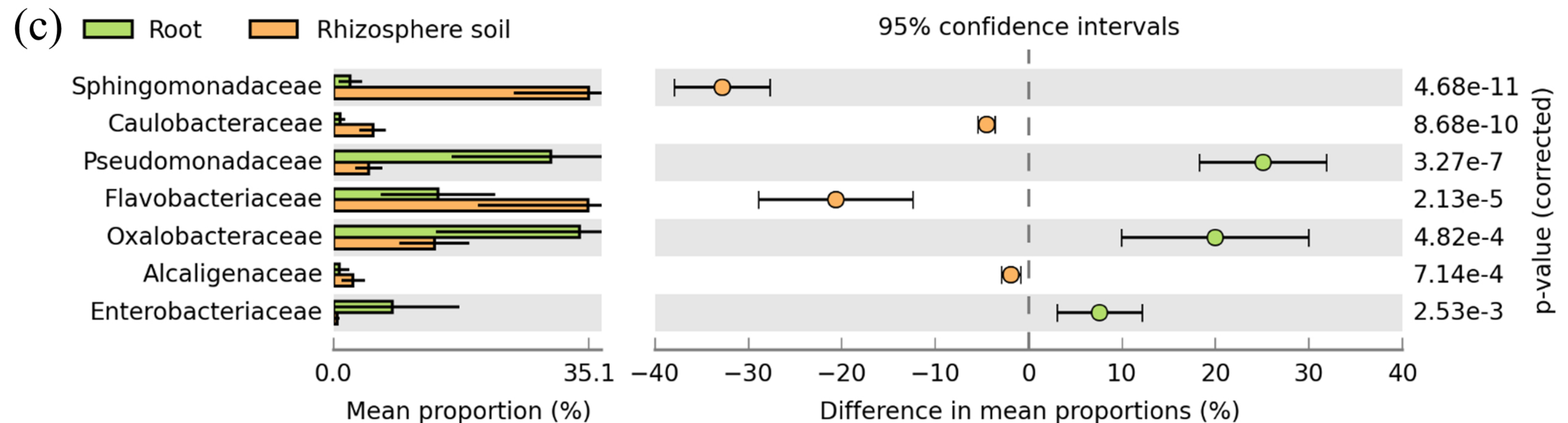

Supplement: Supplemental Information 3 — Welch’s t–tests using STAMP software were conducted to determine whether the differences in abundance between plant compartments were significant. [file peerj-07-6162-s003.pdf]
