# Supplementary material for: Diversity analysis of the rhizospheric and endophytic bacterial communities of Senecio vulgaris L. (Asteraceae) in an invasive range
Source: PeerJ. 2019 Jan 7;6:e6162. doi: 10.7717/peerj.6162 (PMC6327885; doi:10.7717/peerj.6162)
Supplement: Supplemental Information 4 — *contamination was from chloroplasts and mitochondria in the host plants. [file peerj-07-6162-s004.docx]

| Plant compartments | Sampling  Locations | Sample | Before removing contamination ^*^ | | After removing contamination^*^ | |
| --- | --- | --- | --- | --- | --- | --- |
|  |  |  | Total read | Effective reads | Total  read | Effective reads |
| Leaf endosphere | 1 | L1b | 59646 | 57717 | 29094 | 27165 |
|  |  | L1c | 57512 | 55304 | 34938 | 32730 |
|  |  | L1d | 60296 | 59275 | 45406 | 44385 |
|  |  | L1e | 63100 | 61529 | 44751 | 43180 |
|  |  | L1f | 70275 | 68027 | 37372 | 35124 |
|  | 2 | L2a | 64232 | 63045 | 24891 | 23704 |
|  |  | L2b | 55578 | 54462 | 13627 | 12511 |
|  |  | L2c | 70815 | 70048 | 24499 | 23732 |
|  |  | L2d | 61120 | 58071 | 49619 | 46570 |
|  |  | L2e | 59449 | 58429 | 21805 | 20785 |
|  | 3 | L3a | 52990 | 49056 | 32989 | 29055 |
|  |  | L3b | 64351 | 60007 | 43558 | 39214 |
|  |  | L3c | 57478 | 55448 | 39289 | 37259 |
|  |  | L3d | 64748 | 63129 | 52660 | 51041 |
|  |  | L3e | 55267 | 53384 | 38886 | 37003 |
|  | 4 | L4a | 60600 | 51735 | 49299 | 40434 |
|  |  | L4b | 62625 | 60554 | 56986 | 54915 |
|  |  | L4c | 55803 | 52939 | 37592 | 34728 |
|  |  | L4d | 56018 | 52102 | 55150 | 51234 |
| Root endosphere | 1 | R1b | 51326 | 50083 | 51046 | 49803 |
|  |  | R1c | 55351 | 53710 | 54781 | 53140 |
|  |  | R1d | 58841 | 56795 | 58766 | 56720 |
|  |  | R1e | 47782 | 47124 | 47763 | 47105 |
|  |  | R1f | 56965 | 55177 | 56744 | 54956 |
|  | 2 | R2a | 48562 | 46068 | 48465 | 45971 |
|  |  | R2b | 47711 | 45935 | 47313 | 45537 |
|  |  | R2c | 59738 | 56427 | 59073 | 55762 |
|  |  | R2d | 53386 | 50916 | 53027 | 50557 |
|  |  | R2e | 50310 | 48573 | 50053 | 48316 |
|  | 3 | R3a | 54761 | 53910 | 54458 | 53607 |
|  |  | R3b | 62168 | 60484 | 61860 | 60176 |
|  |  | R3c | 58167 | 56573 | 58018 | 56424 |
|  |  | R3d | 50348 | 48529 | 49628 | 47809 |
|  |  | R3e | 60722 | 57868 | 59908 | 57054 |
|  | 4 | R4a | 67055 | 65356 | 66966 | 65267 |
|  |  | R4b | 67277 | 62234 | 67265 | 62222 |
|  |  | R4c | 57208 | 55748 | 57013 | 55553 |
|  |  | R4d | 65222 | 62693 | 64810 | 62281 |
| Rhizosphere | 1 | RS1b | 49906 | 43616 | 49860 | 43570 |
|  |  | RS1c | 54252 | 48033 | 54187 | 47968 |
|  |  | RS1d | 59407 | 52129 | 59359 | 52081 |
|  |  | RS1e | 52587 | 45469 | 52503 | 45385 |
|  |  | RS1f | 48748 | 41843 | 48706 | 41801 |
|  | 2 | RS2a | 62015 | 53974 | 61903 | 53862 |
|  |  | RS2b | 62720 | 54130 | 62658 | 54068 |
|  |  | RS2c | 51903 | 43202 | 51873 | 43172 |
|  |  | RS2d | 50031 | 42989 | 50013 | 42971 |
|  |  | RS2e | 56581 | 48841 | 56577 | 48837 |
|  | 3 | RS3a | 58888 | 51244 | 58787 | 51143 |
|  |  | RS3b | 56175 | 48350 | 56128 | 48303 |
|  |  | RS3c | 59691 | 51075 | 59624 | 51008 |
|  |  | RS3d | 56741 | 49243 | 56674 | 49176 |
|  |  | RS3e | 53765 | 45959 | 53714 | 45908 |
|  | 4 | RS4a | 58514 | 50459 | 58357 | 50302 |
|  |  | RS4b | 55151 | 47863 | 55108 | 47820 |
|  |  | RS4c | 52744 | 46204 | 52699 | 46159 |
|  |  | RS4d | 52206 | 43811 | 52151 | 43756 |
