# Supplementary material for: Diversity analysis of the rhizospheric and endophytic bacterial communities of Senecio vulgaris L. (Asteraceae) in an invasive range
Source: PeerJ. 2019 Jan 7;6:e6162. doi: 10.7717/peerj.6162 (PMC6327885; doi:10.7717/peerj.6162)
Supplement: Supplemental Information 5 [file peerj-07-6162-s005.docx]

| Plant compartments | Sampling  Locations | Sample | Phylum | Class | Order | Family | Genus | Species |
| --- | --- | --- | --- | --- | --- | --- | --- | --- |
|  |  |  | 34^*^ | 60^*^ | 136^*^ | 275^*^ | 458^*^ | 246^*^ |
| Leaf endosphere | 1 | L1b | 27165 | 27156 | 26932 | 26729 | 23461 | 15938 |
|  |  | L1c | 32729 | 32718 | 32337 | 32329 | 27882 | 16631 |
|  |  | L1d | 44385 | 44385 | 44339 | 43504 | 29079 | 19657 |
|  |  | L1e | 43179 | 43176 | 43024 | 42521 | 30244 | 25611 |
|  |  | L1f | 34653 | 34635 | 34007 | 32455 | 27229 | 16218 |
|  | 2 | L2a | 23704 | 23697 | 23662 | 23661 | 20726 | 7942 |
|  |  | L2b | 12511 | 12506 | 12438 | 12409 | 10671 | 4033 |
|  |  | L2c | 23732 | 23732 | 23564 | 22896 | 21217 | 12162 |
|  |  | L2d | 46563 | 46437 | 45579 | 45379 | 42379 | 27177 |
|  |  | L2e | 20784 | 20774 | 20672 | 20667 | 18958 | 8235 |
|  | 3 | L3a | 29031 | 28960 | 28531 | 28472 | 25031 | 10761 |
|  |  | L3b | 39205 | 39150 | 38403 | 38150 | 34974 | 16800 |
|  |  | L3c | 37253 | 37150 | 36727 | 36388 | 33044 | 21199 |
|  |  | L3d | 51038 | 50973 | 50744 | 50110 | 47430 | 15568 |
|  |  | L3e | 36991 | 36928 | 36209 | 36014 | 32585 | 23676 |
|  | 4 | L4a | 40434 | 40295 | 40124 | 39723 | 36145 | 23046 |
|  |  | L4b | 54914 | 54899 | 54614 | 54268 | 46996 | 29176 |
|  |  | L4c | 34715 | 34669 | 34329 | 34191 | 30989 | 15694 |
|  |  | L4d | 51227 | 51174 | 51067 | 51024 | 50044 | 45838 |
| Root endosphere | 1 | R1b | 49762 | 49713 | 49591 | 49406 | 37034 | 8917 |
|  |  | R1c | 53138 | 53079 | 53043 | 52956 | 34387 | 6919 |
|  |  | R1d | 56719 | 56697 | 56674 | 56650 | 30552 | 5713 |
|  |  | R1e | 47104 | 47092 | 47086 | 47081 | 35290 | 11593 |
|  |  | R1f | 54955 | 54922 | 54857 | 54777 | 35777 | 10609 |
|  | 2 | R2a | 45861 | 45815 | 45667 | 45534 | 36251 | 9581 |
|  |  | R2b | 45518 | 45457 | 45330 | 45115 | 36336 | 11110 |
|  |  | R2c | 55752 | 55678 | 55596 | 55157 | 40939 | 12935 |
|  |  | R2d | 50535 | 50454 | 50276 | 50070 | 36575 | 10289 |
|  |  | R2e | 48299 | 48219 | 48026 | 47881 | 43082 | 15518 |
|  | 3 | R3a | 53607 | 53587 | 53548 | 53479 | 33795 | 7206 |
|  |  | R3b | 60156 | 60087 | 60022 | 59888 | 44106 | 7274 |
|  |  | R3c | 56414 | 56347 | 56302 | 56220 | 48435 | 18171 |
|  |  | R3d | 47786 | 47669 | 47583 | 47393 | 32006 | 12994 |
|  |  | R3e | 57033 | 54716 | 54067 | 53698 | 47729 | 29894 |
|  | 4 | R4a | 65258 | 65217 | 65182 | 65148 | 35897 | 15690 |
|  |  | R4b | 62220 | 62208 | 62195 | 62157 | 28762 | 16808 |
|  |  | R4c | 55543 | 55497 | 55355 | 55028 | 31946 | 5718 |
|  |  | R4d | 62255 | 62189 | 61980 | 60840 | 51748 | 12223 |
| Rhizosphere | 1 | RS1b | 43475 | 43044 | 41067 | 34925 | 24010 | 6623 |
|  |  | RS1c | 47913 | 47543 | 45765 | 40115 | 28939 | 7100 |
|  |  | RS1d | 52003 | 51628 | 49942 | 43355 | 31097 | 8232 |
|  |  | RS1e | 45248 | 44686 | 42951 | 35985 | 24071 | 5213 |
|  |  | RS1f | 41711 | 41342 | 39648 | 33431 | 22603 | 5423 |
|  | 2 | RS2a | 53749 | 53307 | 51577 | 43750 | 30840 | 8099 |
|  |  | RS2b | 53941 | 53361 | 51262 | 44750 | 29852 | 7063 |
|  |  | RS2c | 43063 | 42674 | 40540 | 33530 | 23397 | 6465 |
|  |  | RS2d | 42875 | 42504 | 40916 | 33283 | 23795 | 6623 |
|  |  | RS2e | 48722 | 48394 | 46392 | 37999 | 26253 | 7137 |
|  | 3 | RS3a | 51006 | 50636 | 49519 | 44809 | 32276 | 8090 |
|  |  | RS3b | 48244 | 47954 | 46511 | 41670 | 30467 | 7939 |
|  |  | RS3c | 50954 | 50614 | 49078 | 42439 | 30735 | 9017 |
|  |  | RS3d | 49090 | 48297 | 46848 | 40598 | 29011 | 6328 |
|  |  | RS3e | 45786 | 45209 | 43638 | 38425 | 27064 | 5556 |
|  | 4 | RS4a | 50173 | 49736 | 48168 | 43593 | 33229 | 7924 |
|  |  | RS4b | 47795 | 47448 | 46242 | 41859 | 32540 | 11004 |
|  |  | RS4c | 46033 | 45880 | 45065 | 42589 | 34865 | 6227 |
|  |  | RS4d | 43673 | 43177 | 41964 | 38855 | 29189 | 6568 |
