# Supplementary material for: Diversity analysis of the rhizospheric and endophytic bacterial communities of Senecio vulgaris L. (Asteraceae) in an invasive range
Source: PeerJ. 2019 Jan 7;6:e6162. doi: 10.7717/peerj.6162 (PMC6327885; doi:10.7717/peerj.6162)
Supplement: Supplemental Information 6 — Explanation for groups of samples: L, leaf endosphere; R, root endosphere; RS, rhizosphere; 1–4 represent the four locations for sampling. [file peerj-07-6162-s006.docx]

|  | **Estimate** | **Std.Error** | **t value** | **Pr(>\|t\|)** | Signif. codes |
| --- | --- | --- | --- | --- | --- |
| L2 - L1 == 0 | 0.43 | 0.3689 | 1.166 | 0.984 |  |
| L3 - L1 == 0 | 0.3554 | 0.3743 | 0.95 | 0.997 |  |
| L4 - L1 == 0 | -0.1153 | 0.737 | -0.156 | 1 |  |
| R1 - L1 == 0 | 0.218 | 0.4961 | 0.439 | 1 |  |
| R2 - L1 == 0 | 0.5082 | 0.3998 | 1.271 | 0.97 |  |
| R3 - L1 == 0 | -0.2526 | 0.413 | -0.612 | 1 |  |
| R4 - L1 == 0 | -0.5181 | 0.5527 | -0.937 | 0.997 |  |
| **RS1 - L1 == 0** | 4.212 | 0.2876 | 14.646 | <0.001 | *** |
| **RS2 - L1 == 0** | 4.2734 | 0.2913 | 14.669 | <0.001 | *** |
| **RS3 - L1 == 0** | 4.2942 | 0.2753 | 15.597 | <0.001 | *** |
| **RS4 - L1 == 0** | 3.6417 | 0.4122 | 8.834 | <0.001 | *** |
| L3 - L2 == 0 | -0.0746 | 0.3605 | -0.207 | 1 |  |
| L4 - L2 == 0 | -0.5453 | 0.7301 | -0.747 | 1 |  |
| R1 - L2 == 0 | -0.212 | 0.4858 | -0.436 | 1 |  |
| R2 - L2 == 0 | 0.0782 | 0.387 | 0.202 | 1 |  |
| R3 - L2 == 0 | -0.6826 | 0.4006 | -1.704 | 0.819 |  |
| R4 - L2 == 0 | -0.948 | 0.5435 | -1.744 | 0.797 |  |
| **RS1 - L2 == 0** | 3.782 | 0.2695 | 14.034 | <0.001 | *** |
| **RS2 - L2 == 0** | 3.8434 | 0.2735 | 14.054 | <0.001 | *** |
| **RS3 - L2 == 0** | 3.8642 | 0.2564 | 15.073 | <0.001 | *** |
| **RS4 - L2 == 0** | 3.2117 | 0.3998 | 8.033 | <0.001 | *** |
| L4 - L3 == 0 | -0.4707 | 0.7329 | -0.642 | 1 |  |
| R1 - L3 == 0 | -0.1374 | 0.4899 | -0.28 | 1 |  |
| R2 - L3 == 0 | 0.1528 | 0.3921 | 0.39 | 1 |  |
| R3 - L3 == 0 | -0.608 | 0.4056 | -1.499 | 0.911 |  |
| R4 - L3 == 0 | -0.8734 | 0.5472 | -1.596 | 0.872 |  |
| **RS1 - L3 == 0** | 3.8566 | 0.2768 | 13.934 | <0.001 | *** |
| **RS2 - L3 == 0** | 3.918 | 0.2807 | 13.96 | <0.001 | *** |
| **RS3 - L3 == 0** | 3.9388 | 0.264 | 14.918 | <0.001 | *** |
| **RS4 - L3 == 0** | 3.2863 | 0.4047 | 8.119 | <0.001 | *** |
| R1 - L4 == 0 | 0.3333 | 0.8019 | 0.416 | 1 |  |
| R2 - L4 == 0 | 0.6235 | 0.7462 | 0.836 | 0.999 |  |
| R3 - L4 == 0 | -0.1373 | 0.7534 | -0.182 | 1 |  |
| R4 - L4 == 0 | -0.4027 | 0.8381 | -0.481 | 1 |  |
| **RS1 - L4 == 0** | 4.3273 | 0.6926 | 6.248 | <0.001 | *** |
| **RS2 - L4 == 0** | 4.3887 | 0.6942 | 6.322 | <0.001 | *** |
| **RS3 - L4 == 0** | 4.4095 | 0.6876 | 6.413 | <0.001 | *** |
| **RS4 - L4 == 0** | 3.757 | 0.7529 | 4.99 | <0.001 | *** |
| R2 - R1 == 0 | 0.2902 | 0.5097 | 0.569 | 1 |  |
| R3 - R1 == 0 | -0.4706 | 0.5201 | -0.905 | 0.998 |  |
| R4 - R1 == 0 | -0.736 | 0.6367 | -1.156 | 0.985 |  |
| **RS1 - R1 == 0** | 3.994 | 0.4274 | 9.346 | <0.001 | *** |
| **RS2 - R1 == 0** | 4.0554 | 0.4299 | 9.434 | <0.001 | *** |
| **RS3 - R1 == 0** | 4.0762 | 0.4192 | 9.723 | <0.001 | *** |
| **RS4 - R1 == 0** | 3.4237 | 0.5195 | 6.591 | <0.001 | *** |
| R3 - R2 == 0 | -0.7608 | 0.4292 | -1.772 | 0.781 |  |
| R4 - R2 == 0 | -1.0263 | 0.5649 | -1.817 | 0.754 |  |
| **RS1 - R2 == 0** | 3.7038 | 0.3104 | 11.933 | <0.001 | *** |
| **RS2 - R2 == 0** | 3.7652 | 0.3139 | 11.996 | <0.001 | *** |
| **RS3 - R2 == 0** | 3.786 | 0.2991 | 12.659 | <0.001 | *** |
| **RS4 - R2 == 0** | 3.1335 | 0.4284 | 7.314 | <0.001 | *** |
| R4 - R3 == 0 | -0.2655 | 0.5744 | -0.462 | 1 |  |
| **RS1 - R3 == 0** | 4.4646 | 0.3273 | 13.641 | <0.001 | *** |
| **RS2 - R3 == 0** | 4.526 | 0.3306 | 13.691 | <0.001 | *** |
| **RS3 - R3 == 0** | 4.5468 | 0.3166 | 14.362 | <0.001 | *** |
| **RS4 - R3 == 0** | 3.8943 | 0.4408 | 8.834 | <0.001 | *** |
| **RS1 - R4 == 0** | 4.7301 | 0.492 | 9.615 | <0.001 | *** |
| **RS2 - R4 == 0** | 4.7915 | 0.4942 | 9.696 | <0.001 | *** |
| **RS3 - R4 == 0** | 4.8122 | 0.4849 | 9.924 | <0.001 | *** |
| **RS4 - R4 == 0** | 4.1597 | 0.5738 | 7.25 | <0.001 | *** |
| RS2 - RS1 == 0 | 0.0614 | 0.1463 | 0.42 | 1 |  |
| RS3 - RS1 == 0 | 0.0822 | 0.111 | 0.74 | 1 |  |
| RS4 - RS1 == 0 | -0.5703 | 0.3263 | -1.748 | 0.794 |  |
| RS3 - RS2 == 0 | 0.0208 | 0.1204 | 0.173 | 1 |  |
| RS4 - RS2 == 0 | -0.6317 | 0.3296 | -1.917 | 0.69 |  |
| RS4 - RS3 == 0 | -0.6525 | 0.3155 | -2.068 | 0.588 |  |
