# Supplementary material for: Diversity analysis of the rhizospheric and endophytic bacterial communities of Senecio vulgaris L. (Asteraceae) in an invasive range
Source: PeerJ. 2019 Jan 7;6:e6162. doi: 10.7717/peerj.6162 (PMC6327885; doi:10.7717/peerj.6162)
Supplement: Supplemental Information 7 — /= unidentified taxa. [file peerj-07-6162-s007.docx]

| **OTU NO.** | **Phylum** | **Class** | **Order** | **Family** | **Genus** | **Species** | **Relative**  **Abundance** | **Relative**  **Frequency** |
| --- | --- | --- | --- | --- | --- | --- | --- | --- |
| OTU_77 | Actinobacteria | unidentified | Corynebacteriales | Corynebacteriaceae | Corynebacterium_1 | tuberculostearicum | 0.004 | 0.84 |
| OTU_31 | Actinobacteria | unidentified | Corynebacteriales | Mycobacteriaceae | Mycobacterium | / | 0.010 | 0.79 |
| OTU_113 | Actinobacteria | unidentified | Micrococcales | Brevibacteriaceae | Brevibacterium | casei | 0.004 | 0.74 |
| OTU_38 | Actinobacteria | unidentified | Micrococcales | Micrococcaceae | Kocuria | rosea | 0.010 | 0.79 |
| OTU_28 | Actinobacteria | unidentified | Propionibacteriales | Propionibacteriaceae | Propionibacterium | / | 0.019 | 1.00 |
| OTU_8 | Bacteroidetes | Flavobacteriia | Flavobacteriales | Flavobacteriaceae | Chryseobacterium | shigense | 0.028 | 1.00 |
| OTU_16 | Bacteroidetes | Flavobacteriia | Flavobacteriales | Flavobacteriaceae | Flavobacterium | pectinovorum | 0.003 | 0.74 |
| OTU_24 | Firmicutes | Bacilli | Bacillales | Bacillaceae | Bacillus | / | 0.014 | 0.74 |
| OTU_10 | Firmicutes | Bacilli | Bacillales | Family_XII | Exiguobacterium | sibiricum | 0.070 | 1.00 |
| OTU_60 | Firmicutes | Bacilli | Bacillales | Staphylococcaceae | Staphylococcus | epidermidis | 0.008 | 1.00 |
| OTU_4 | Proteobacteria | Alphaproteobacteria | Caulobacterales | Caulobacteraceae | Brevundimonas | diminuta | 0.187 | 1.00 |
| OTU_1340 | Proteobacteria | Alphaproteobacteria | Caulobacterales | Caulobacteraceae | Brevundimonas | / | 0.003 | 1.00 |
| OTU_96 | Proteobacteria | Alphaproteobacteria | Rhizobiales | Bradyrhizobiaceae | Bosea | thiooxidans | 0.004 | 0.95 |
| OTU_36 | Proteobacteria | Alphaproteobacteria | Rhizobiales | Bradyrhizobiaceae | Bradyrhizobium | elkanii | 0.004 | 0.79 |
| OTU_1507 | Proteobacteria | Alphaproteobacteria | Rhizobiales | Rhizobiaceae | Ensifer | / | 0.011 | 1.00 |
| OTU_524 | Proteobacteria | Alphaproteobacteria | Sphingomonadales | Sphingomonadaceae | Sphingomonas | aerolata | 0.024 | 0.74 |
| OTU_13 | Proteobacteria | Alphaproteobacteria | Sphingomonadales | Sphingomonadaceae | Sphingomonas | faeni | 0.021 | 0.89 |
| OTU_20 | Proteobacteria | Alphaproteobacteria | Sphingomonadales | Sphingomonadaceae | Sphingomonas | / | 0.014 | 1.00 |
| OTU_33 | Proteobacteria | Alphaproteobacteria | Sphingomonadales | Sphingomonadaceae | Sphingomonas | echinoides | 0.006 | 0.95 |
| OTU_4308 | Proteobacteria | Alphaproteobacteria | / | / | / | / | 0.005 | 1.00 |
| OTU_6 | Proteobacteria | Betaproteobacteria | Burkholderiales | Alcaligenaceae | / | / | 0.052 | 1.00 |
| OTU_30 | Proteobacteria | Betaproteobacteria | Burkholderiales | Comamonadaceae | Pelomonas | / | 0.008 | 1.00 |
| OTU_18 | Proteobacteria | Betaproteobacteria | Burkholderiales | Comamonadaceae | Variovorax | paradoxus | 0.008 | 0.84 |
| OTU_5 | Proteobacteria | Betaproteobacteria | Burkholderiales | Oxalobacteraceae | Duganella | / | 0.019 | 0.95 |
| OTU_61 | Proteobacteria | Betaproteobacteria | Burkholderiales | Oxalobacteraceae | Massilia | / | 0.003 | 0.79 |
| OTU_23 | Proteobacteria | Betaproteobacteria | Burkholderiales | Oxalobacteraceae | Massilia | / | 0.003 | 0.84 |
| OTU_3 | Proteobacteria | Betaproteobacteria | Burkholderiales | Oxalobacteraceae | / | / | 0.013 | 1.00 |
| OTU_1514 | Proteobacteria | Betaproteobacteria | Burkholderiales | Oxalobacteraceae | / | / | 0.005 | 0.84 |
| OTU_17 | Proteobacteria | Gammaproteobacteria | Enterobacteriales | Enterobacteriaceae | / | / | 0.004 | 0.84 |
| OTU_2869 | Proteobacteria | Gammaproteobacteria | Enterobacteriales | Enterobacteriaceae | / | / | 0.003 | 0.95 |
| OTU_29 | Proteobacteria | Gammaproteobacteria | Pseudomonadales | Moraxellaceae | Acinetobacter | / | 0.010 | 0.95 |
| OTU_7 | Proteobacteria | Gammaproteobacteria | Pseudomonadales | Pseudomonadaceae | Pseudomonas | / | 0.054 | 1.00 |
| OTU_9 | Proteobacteria | Gammaproteobacteria | Pseudomonadales | Pseudomonadaceae | Pseudomonas | viridiflava | 0.050 | 0.95 |
| OTU_14 | Proteobacteria | Gammaproteobacteria | Pseudomonadales | Pseudomonadaceae | Pseudomonas | / | 0.014 | 0.89 |
| OTU_4063 | Proteobacteria | Gammaproteobacteria | Pseudomonadales | Pseudomonadaceae | Pseudomonas | / | 0.006 | 0.84 |
| OTU_1674 | Proteobacteria | Gammaproteobacteria | Pseudomonadales | Pseudomonadaceae | / | / | 0.003 | 0.74 |
