# Supplementary material for: Diversity analysis of the rhizospheric and endophytic bacterial communities of Senecio vulgaris L. (Asteraceae) in an invasive range
Source: PeerJ. 2019 Jan 7;6:e6162. doi: 10.7717/peerj.6162 (PMC6327885; doi:10.7717/peerj.6162)
Supplement: Supplemental Information 8 — /= unidentified taxa. [file peerj-07-6162-s008.docx]

| **OTU NO.** | **Phylum** | **Class** | **Order** | **Family** | **Genus** | **Species** | **Relative**  **Abundance** | **Relative**  **Frequency** |
| --- | --- | --- | --- | --- | --- | --- | --- | --- |
| OTU_12 | Proteobacteria | Alphaproteobacteria | Rhizobiales | Rhizobiaceae | Ensifer | / | 0.021 | 0.68 |
| OTU_11 | Proteobacteria | Alphaproteobacteria | Rhizobiales | Rhizobiaceae | Rhizobium | leguminosarum | 0.032 | 0.84 |
| OTU_13 | Proteobacteria | Alphaproteobacteria | Sphingomonadales | Sphingomonadaceae | Sphingomonas | faeni | 0.007 | 0.95 |
| OTU_8 | Bacteroidetes | Flavobacteriia | Flavobacteriales | Flavobacteriaceae | Chryseobacterium | shigense | 0.017 | 1.00 |
| OTU_16 | Bacteroidetes | Flavobacteriia | Flavobacteriales | Flavobacteriaceae | Flavobacterium | pectinovorum | 0.042 | 1.00 |
| OTU_2051 | Bacteroidetes | Flavobacteriia | Flavobacteriales | Flavobacteriaceae | Flavobacterium | / | 0.019 | 1.00 |
| OTU_885 | Bacteroidetes | Flavobacteriia | Flavobacteriales | Flavobacteriaceae | Flavobacterium | / | 0.013 | 1.00 |
| OTU_40 | Bacteroidetes | Flavobacteriia | Flavobacteriales | Flavobacteriaceae | Flavobacterium | / | 0.010 | 1.00 |
| OTU_22 | Proteobacteria | Alphaproteobacteria | Rhizobiales | Rhizobiaceae | Rhizobium | / | 0.014 | 1.00 |
| OTU_34 | Proteobacteria | Alphaproteobacteria | Sphingomonadales | Sphingomonadaceae | Sphingobium | / | 0.005 | 1.00 |
| OTU_6 | Proteobacteria | Betaproteobacteria | Burkholderiales | Alcaligenaceae | / | / | 0.005 | 1.00 |
| OTU_351 | Proteobacteria | Betaproteobacteria | Burkholderiales | Comamonadaceae | Acidovorax | / | 0.005 | 1.00 |
| OTU_18 | Proteobacteria | Betaproteobacteria | Burkholderiales | Comamonadaceae | Variovorax | paradoxus | 0.008 | 1.00 |
| OTU_39 | Proteobacteria | Betaproteobacteria | Burkholderiales | Comamonadaceae | / | / | 0.006 | 1.00 |
| OTU_5 | Proteobacteria | Betaproteobacteria | Burkholderiales | Oxalobacteraceae | Duganella | / | 0.062 | 1.00 |
| OTU_107 | Proteobacteria | Betaproteobacteria | Burkholderiales | Oxalobacteraceae | Massilia | / | 0.018 | 1.00 |
| OTU_2733 | Proteobacteria | Betaproteobacteria | Burkholderiales | Oxalobacteraceae | Massilia | / | 0.008 | 1.00 |
| OTU_4758 | Proteobacteria | Betaproteobacteria | Burkholderiales | Oxalobacteraceae | Massilia | / | 0.005 | 1.00 |
| OTU_3 | Proteobacteria | Betaproteobacteria | Burkholderiales | Oxalobacteraceae | / | / | 0.167 | 1.00 |
| OTU_27 | Proteobacteria | Betaproteobacteria | Methylophilales | Methylophilaceae | Methylophilus | / | 0.009 | 1.00 |
| OTU_1112 | Proteobacteria | Betaproteobacteria | Methylophilales | Methylophilaceae | Methylotenera | / | 0.009 | 1.00 |
| OTU_547 | Proteobacteria | Betaproteobacteria | Methylophilales | Methylophilaceae | / | / | 0.006 | 1.00 |
| OTU_17 | Proteobacteria | Gammaproteobacteria | Enterobacteriales | Enterobacteriaceae | / | / | 0.034 | 1.00 |
| OTU_2869 | Proteobacteria | Gammaproteobacteria | Enterobacteriales | Enterobacteriaceae | / | / | 0.026 | 1.00 |
| OTU_9 | Proteobacteria | Gammaproteobacteria | Pseudomonadales | Pseudomonadaceae | Pseudomonas | viridiflava | 0.073 | 1.00 |
| OTU_7 | Proteobacteria | Gammaproteobacteria | Pseudomonadales | Pseudomonadaceae | Pseudomonas | / | 0.094 | 1.00 |
| OTU_14 | Proteobacteria | Gammaproteobacteria | Pseudomonadales | Pseudomonadaceae | Pseudomonas | / | 0.028 | 1.00 |
| OTU_3396 | Proteobacteria | Gammaproteobacteria | Pseudomonadales | Pseudomonadaceae | Pseudomonas | / | 0.019 | 1.00 |
| OTU_4063 | Proteobacteria | Gammaproteobacteria | Pseudomonadales | Pseudomonadaceae | Pseudomonas | / | 0.019 | 1.00 |
| OTU_19 | Proteobacteria | Gammaproteobacteria | Xanthomonadales | Xanthomonadaceae | Stenotrophomonas | / | 0.017 | 1.00 |
